# Interspecies relationships of natural amoebae and bacteria with *C. elegans* create environments propitious for multigenerational diapause

**DOI:** 10.1128/msystems.01566-24

**Published:** 2025-03-20

**Authors:** Marcela Serey, Esteban Retamales, Gabriel Ibañez, Gonzalo Riadi, Patricio Orio, Juan P. Castillo, Andrea Calixto

**Affiliations:** 1 Centro Interdisciplinario de Neurociencia de Valparaíso, Instituto de Neurociencia, Facultad de Ciencias, Universidad de Valparaíso, Valparaiso, Valparaíso Region, Chile; 2 Universidad de Valparaíso 28068 , Valparaiso, Chile; 3 Department of Bioinformatics, ANID–Millennium Science Initiative Program Millennium Nucleus of Ion Channels-Associated Diseases (MiNICAD), Center for Bioinformatics, Simulation and Modeling (CBSM), Faculty of Engineering, University of Talca 28066 , Talca, Maule Region, Chile; Max Planck Institute for Marine Microbiology, Bremen, Germany

**Keywords:** natural environments, amoebae, microbiota, behavior, diapause, multigenerational, RNAi, interspecies communication

## Abstract

The molecular and physical communication within the microscopic world underpins the entire web of life as we know it. However, how organisms, such as bacteria, amoebae, and nematodes—all ubiquitous—interact to sustain their ecological niches, particularly how their associations generate and influence behavior, remains largely unknown. In this study, we developed a framework to examine long-term interactions between microbes and animals. From soil samples collected in a temperate, semi-arid climate, we isolated culturable bacterial genera, including *Comamonas*, *Stenotrophomonas*, *Chryseobacterium*, and *Rhodococcus*, as well as the amoeba, *Tetramitus*. This microbial ensemble was fed to the nematode *C. elegans* in experiments spanning over 20 nematode generations to assess developmental rate, dauer entry, fertility, and feeding behavior. Our findings reveal that microbes and nematodes create a stable environment where no species are exhausted, and where nematodes enter diapause after several generations. We have termed this phenomenon dauer formation on naturally derived ensembles (DaFNE). DaFNE occurs across a range of optimal temperatures, from 15°C to 25°C, and is dependent on the nematode’s pheromone biosynthesis pathway. The phenomenon intensifies with each passing generation, exhibiting both strong intergenerational and transgenerational effects. Moreover, the RNA interference (RNAi) pathway—both systemic and cell-autonomous—is essential for initiating DaFNE, while heritable RNAi effectors are required for its transgenerational effects. These findings indicate that RNA-mediated communication plays a critical role in bacterially induced behaviors in natural environments.

**IMPORTANCE:**

Microscopic nematodes are the most abundant multicellular animals on Earth, which implies they have evolved highly successful relationships with their associated microbiota. However, little is known about how nematode behavior is influenced within complex ecosystems where multiple organisms interact. In this study, we used four bacteria and an amoeba from a natural ecosystem to explore behavioral responses in the nematode *Caenorhabditis elegans* over an 8 week period. The most striking finding was the nematodes’ commitment to a form of hibernation known as diapause. We have termed this phenomenon dauer formation on naturally derived ensembles (DaFNE). Our results suggest that nematodes in nature may frequently enter hibernation as a result of communication with their microbial partners. DaFNE requires the production of nematode pheromones, as well as the RNA interference pathway, indicating that the RNA communication between nematodes and their microbiota may play a critical role. Interestingly, at higher temperatures, fewer animals are needed to trigger DaFNE, suggesting that a mild increase in temperature may promote diapause in natural environments without causing stress to the animals.

## INTRODUCTION

The relationship between ubiquitous microscopic organisms, such as protists, bacteria, and nematodes, is fundamental to the health of ecosystems and the sustainability of the biosphere (1, 2). Despite this, the ways in which these organisms interact to maintain their ecological niches remain largely understudied, partly due to the complexity of the variables involved in natural environments. A critical aspect of niche stability is the interaction between microfauna and nematodes, as microbial communities play a key role in shaping nematode behavior. Although several aspects of wild nematode biology have been explored in the context of their natural bacterial communities (3, 4), the role of amoebae in these interactions has been notably absent from most studies on nematode–bacteria dynamics (5). *Caenorhabditis elegans* is an ideal representative species of the broader Nematoda class for studying behavioral responses to biotic variation (6–10). One key adaptation of nematodes to environmental changes is their ability to enter quiescence (11). In the laboratory, *C. elegans* enters diapause, forming dauer larvae in response to crowding, food scarcity, elevated temperatures, or bacterial pathogenesis (12–15). However, while dauers are common in natural settings, the specific cues that trigger dauer entry in these environments remain unknown (16, 17). In this study, we found that a microbial ensemble consisting of *Comamonas*, *Rhodococcus*, *Chryseobacterium*, and *Stenotrophomonas* and the amoeba *Tetramitus* induces *C. elegans* to enter diapause without causing pathogenesis (18, 19). Moreover, in these ensembles, none of the species were depleted over the course of long-term 8 week experiments, suggesting that dauer formation on naturally derived ensembles (DaFNE) plays a role in regulating ecosystem sustainability.

Adaptive responses of *C. elegans* to bacteria that cause pathogenesis (15, 20) use effectors of the RNA interference machinery at different cellular and mechanistic levels. RNAi can be transmitted to the offspring (21, 22) and also produce transgenerational epigenetic inheritance (23, 24). We show that mutations in *sid-2*, essential for dsRNA entry to the intestinal cells (25, 26), and *rde-1*, needed for processing of exogenous dsRNA (27, 28), are required for DaFNE. Furthermore, the Argonaute protein HRDE-1, which is required for heritable RNAi (29), is essential for DaFNE’s transgenerational inheritance, highlighting a broader role for RNAi in interspecies interactions.

## RESULTS

### A natural microbial ensemble of bacteria and amoebae to study long-term relationships with *C. elegans*

**Fig 1:**
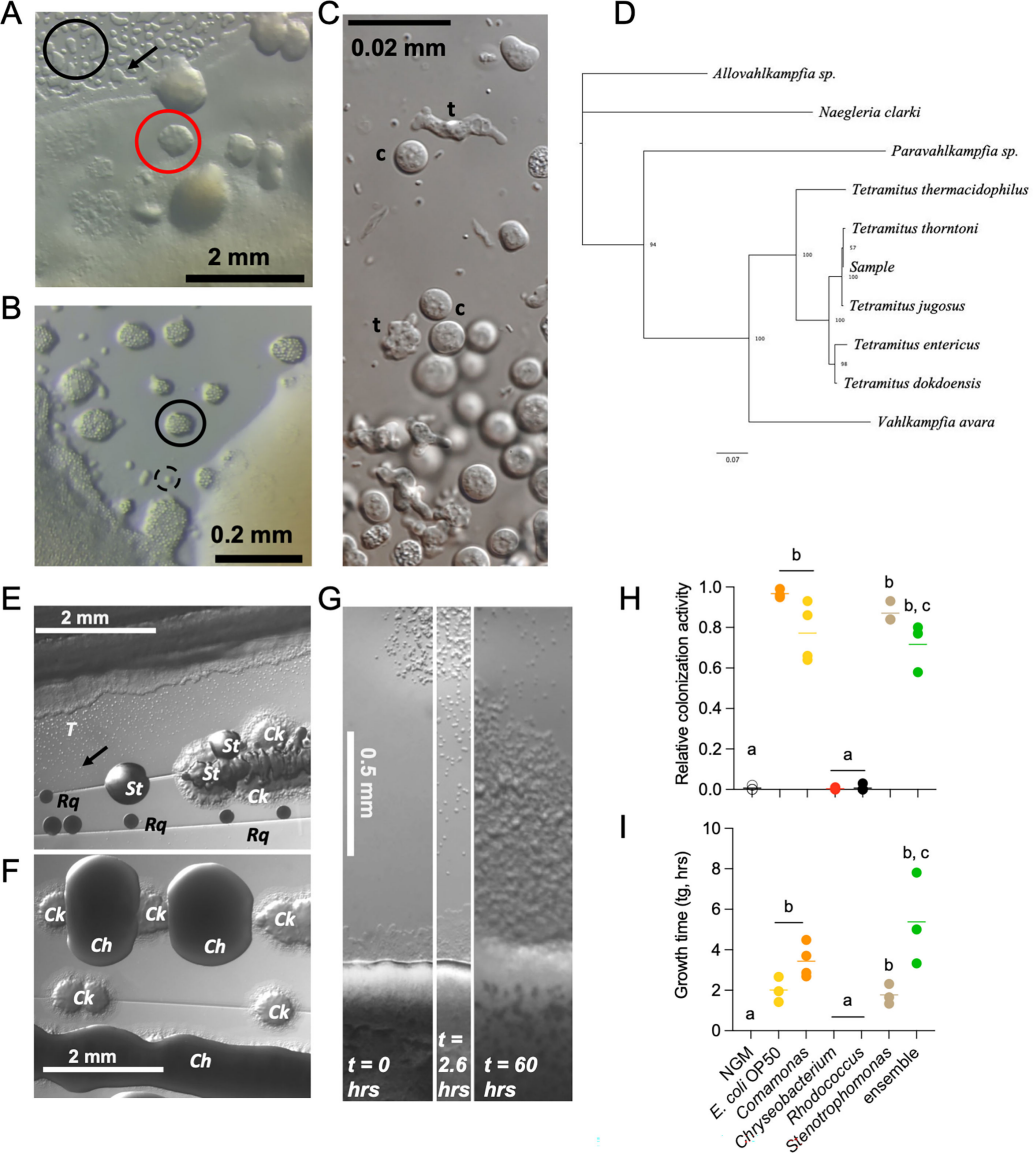
Identification, phylogeny, and growth behavior of *Tetramitus* amoebae in a natural microbial ensemble. (A) Natural microbial ensemble growing on agar-NGM medium, displaying distinct structural features. High-contrast irregular structures on the agar correspond to large patches of *Tetramitus* cysts (black circle). High-contrast round objects (red circle) represent colony-like pools of bacteria and *Tetramitus* trophozoites, as observed in time-lapse video microscopy (Movie S1). A single amoeba cyst is marked with a black arrow. (B) Microbial ensemble growing on an LB plate, showing patches of *Tetramitus* cysts (solid circle) and a moving trophozoite (dashed circle). *Tetramitus* preferentially accumulates in specific regions of the bacterial ensemble (left) while avoiding other areas (right). These separated sectors remained distinct and continued growing for several days post-inoculation. (C) Differential interference contrast (DIC) microscopy image of *Tetramitus* cysts (c) and trophozoites (t) isolated from the original soil sample. (D) Maximum-likelihood consensus phylogenetic inference for the *Tetramitus* sample and five related *Tetramitus* species based on the ribosomal RNA sequence (18S and 5.8S, including ITS1 and ITS2 rRNAs). Node labels indicate bootstrap values from 1,000 iterations. The tree is rooted in *Allovahlkampfia* sp., with *Naegleria clarki*, *Paravahlkampfia* sp., and *Vahlkampfia avara* as outgroups. (E) Colony isolation from the natural soil sample on LB medium revealing distinct microbial species, including *Rhodococcus* (Rq), *Stenotrophomonas* (St), *Comamonas* (Ck), and *Tetramitus* cysts (T). (F) Colony isolation from a different soil subculture containing *Comamonas* (*Ck*) and *Chryseobacterium* (*Ch*). (G) Time-lapse experiment tracking *Tetramitus* amoebae colonization of the bacterial ensemble lawn. Initially, (t = 0 h, left), *Tetramitus* cysts are present at the top of the panel, with the bacterial ensemble at the bottom. By 2.6 h (center), some amoebae have excysted and begun exploring the substrate, reaching the bacterial lawn. By 60 h post-inoculation (right), the amoebae have extensively colonized the bacterial lawn, leaving behind cysts and trophozoites across the recorded area. (H, I) Relative colonization activity (H) and growth time (I) of *Tetramitus* in different bacterial lawns analyzed through cumulative activity over time (Fig. S2). Amoebae failed to colonize NGM, *Chryseobacterium,* and *Rhodococcus*, where colonization fractions approached zero, and growth times exceeded 100 h. These conditions (letter a) significantly differed from all others. A full parametric description is available in Table 2. Same letters denote no statistical differences, while different letters indicate statistically significant differences. Each experiment included three technical triplicates and at least three biological replicates. Complete statistical analyses are detailed in Data set S1.

To establish a multi-organismal framework, we collected a soil sample from a natural urban ecosystem and identified the culturable species of amoebae and bacteria. As a representing species of the superabundant nematodes (2, 30–32), we chose the pioneer organism *C. elegans,* which is ideal to study behavioral outputs in response to biotic environments and their molecular mechanisms (3, 4). We isolated the microbes by mixing sterile water with the soil sample and placing the supernatant on solid agars. Individual bacteria were isolated by successive passages in solid Luria Bertani (LB) media, whereas amoebae were maintained on nematode growth media (NGM) plates (Fig. 1A and Movie S1). Wild nematodes were also found but were not cultivated. The original culture of microbes separated into two subcultures, with amoeba enriched in one of them (Fig. 1B).

Bacteria were identified by full-length ribosomal 16S sequencing as gram-negative species of the genera *Comamonas* (Fig. S1A), *Stenotrophomonas* (Fig. S1B), and *Chryseobacterium* (Fig. S1C), and the gram-positive *Rhodococcus* (Fig. S1D). We found the amoebae in two states: cysts, which are immobile, and trophozoites that move freely on the substrate (Fig. 1C). We classified the amoebae isolates as *Tetramitus* by 18S and internal transcribed spacer (ITS) sequencing. The final tree shown (Fig. 1D) was arbitrarily chosen among all resulting tree searches since all have the same topology and support values. This tree is compatible with previous trees published (33, 34). The sample is shown to be related to *T. thorntoni* and *T. jugosus* according to this phylogenetic tree, which was reconstructed from 18S and ITS sequences. Trees using only ITS sequences (218 informative positions) or only 18S (1,227 informative positions) for phylogenetic reconstruction show a similar relationship. These trees suggest that both *Tetramitus* and the sample species are closely related. The support values in the tree inform us that it is not possible to discriminate either species; hence, we will name the amoeba by the genus name. The species forming the ensemble are described in Table 1.

**TABLE 1:** Identification of the ensemble

Genus	Possible species
Bacteria (16S, gram-negative)	
*Comamonas*	*Comamonas koreensis*
*Stenotrophomonas*	*S. lactitubi*, *S. maltophilia*
*Chryseobacterium*	*C. oleae*, *C. cucumeris*, *C. indologenes*, *C. culicis*
Bacteria (16S, gram-positive): *Rhodococcus*	*R. qingshengii*, *R. jialingiae*
Amoebae (18S, ITS): *Tetramitus*	*Tetramitus jugosus*, *Tetramitus thorntoni*

*Tetramitus* was found in ensembles containing *Comamonas*, *Stenotrophomonas*, and to a lesser extent, *Rhodococcus* (Fig. 1E) but was absent in the mix containing *Chryseobacterium* (Fig. 1F). This indicates that certain bacteria may be more supportive of *Tetramitus* growth. We exposed inocula of *Tetramitus* cysts to the ensemble, monocultures, and *E. coli* OP50 (the standard bacterial food of *C. elegans* in the laboratory) recording their activity patterns through time-lapse acquisition (Fig. 1G; Movie S2). Activity was quantified by subtracting consecutive above background levels (Fig. S2A; see Materials and Methods). The cumulative sum of pixel intensity change over time was expressed as *cumulative activity* (*CA*). Across all experiments, *CA* initially increased following a mono-exponential time course that plateaued before 24 h (Fig. S2B). This initial activity reflects amoebae cysts transitioning into trophozoites, exploring the substrate until they either begin feeding on bacteria or become cysts (Movie S2). We refer to the amplitude of this first plateau as the cumulative inoculum activity (cIA; Fig. S2C). For bacteria supporting *Tetramitus* growth and proliferation (*E. coli* OP50, *Comamonas, Stenotrophomonas*, and the ensemble), a second increase in activity was observed as amoebae propagated on the bacterial lawn (Fig. 1G). We termed this second phase the cumulative colonization activity (cCA), which was absent with *Chryseobacterium* and *Rhodococcus*, or unseeded NGM plates that did not support growth (Fig. S2B). This absence of *Tetramitus* activity suggests that these two bacteria may be either toxic or inedible for the amoebae or both.

Equation 1 (Materials and Methods; Fig. S2C) was used as an *ad-hoc* model to fit experimental data and extract parametrized information (Table 2). Cumulative inoculum activities showed similar characteristics across all bacteria tested, with no statistically significant differences in amplitude (Fig. S2D), excystation time (tx; Fig. S2E), or exploration time (tp; Fig. S2E). This indicates that excystation and exploration are not significantly influenced by chemical cues released by the tested bacteria. In *E. coli* OP50, relative colonization activity [cCA/(cCA *+* cIA)] was both higher (Fig. 1H) and faster (Fig. 1I) than in any other bacteria. Conversely, amoeba proliferated more slowly in the ensemble with the lowest relative colonization activity (Fig. 1H and I; Fig. S2B). These reduced parameters in the ensemble are likely due to the presence of *Chryseobacterium* and *Rhodococcus*, which do not support *Tetramitus* growth.

**TABLE 2:** Parameter details of amoebae tracking

Parameter	NGM	*E. coli* OP50	*Comamonas*	*Chryseobacterium*	*Rhodococcus*	*Stenotrophomonas*	Ensemble
Cumulative inoculum activity (cIA) (AU)	88.9 ± 70.4	60.3 ± 44.6	69.1 ± 26.6	95.2 ± 47.7	102.3 ± 53.2	149.8 ± 53.2	343.5 ± 190.8
Relative colonization activity [cCA/(cCA + cIA)]	0.0 ± 0.0	1.0 ± 0.0	0.8 ± 0.1	0.0 ± 0.0	0.0 ± 0.0	0.9 ± 0.0	0.7 ± 0.1
Excystation delay (tx) (h)	4.6 ± 0.8	5.6 ± 1.8	3.5 ± 0.6	4.0 ± 0.2	2.6 ± 0.4	2.8 ± 0.2	2.6 ± 0.7
Exploration time (tp) (h)	9.3 ± 4.2	9.6 ± 2.3	9.0 ± 0.8	5.2 ± 0.6	9.6 ± 4.2	9.1 ± 2.1	5.5 ± 0.8
Colonization time (tc) (h)	>200	52.7 ± 3.6	47.0 ± 2.9	>200	>200	67.7 ± 6.4	40.1 ± 12.2
Growth time (tg) (h)	>200	2.0 ± 0.4	3.4 ± 0.3	>200	>200	1.8 ± 0.3	5.4 ± 1.3
*N*	3	3	4	3	4	3	3

### *C. elegans* feeds and develops in the ensemble

**Fig 2:**
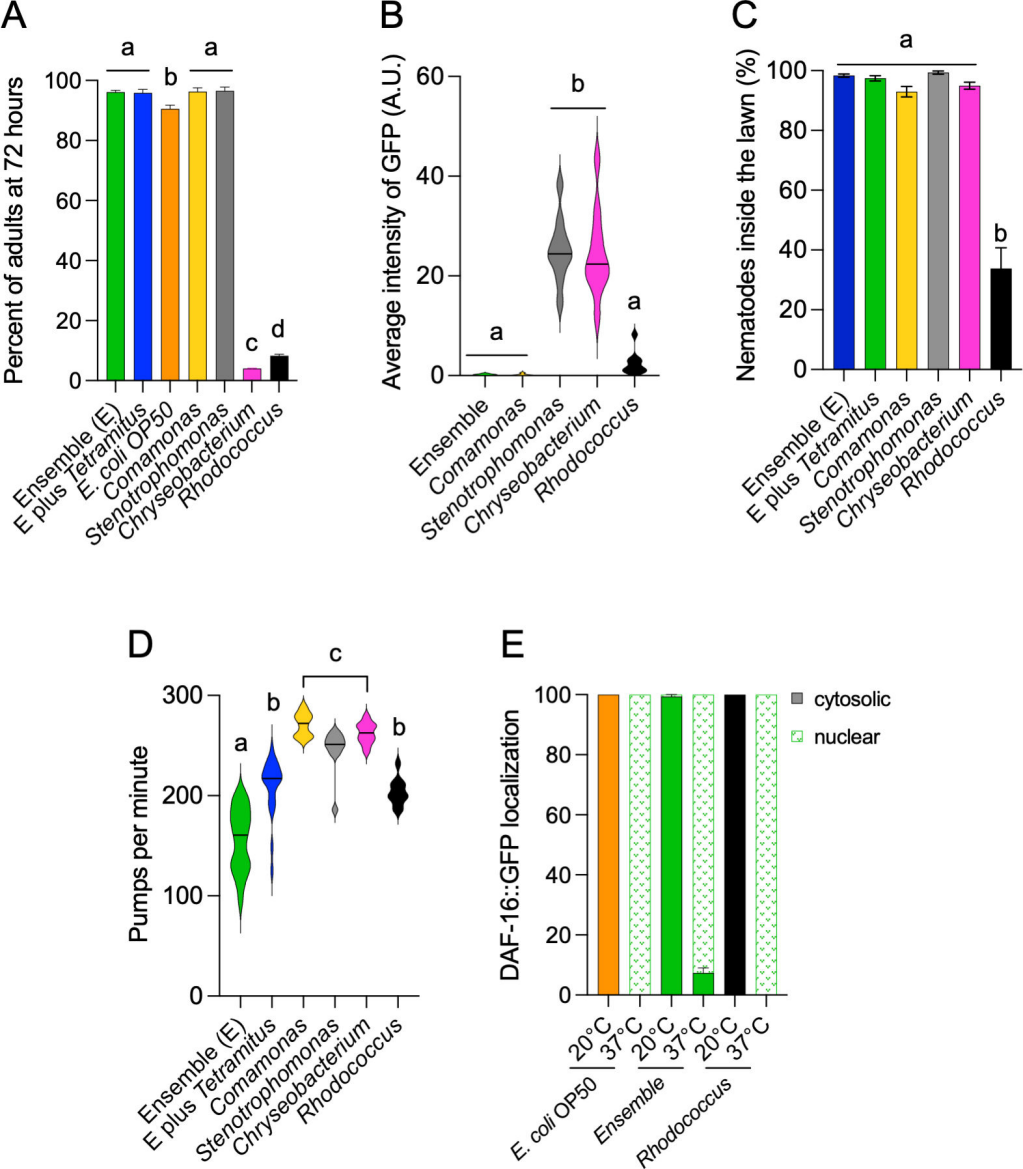
*C. elegans* physiological and behavioral responses to the natural microbial ensemble (A). Percentage of adult *C. elegans* after 72 h of feeding on either the microbial ensemble or individual bacterial monocultures. (B) Intestinal vitamin B12 sensing in nematodes measured as arbitrary units (AU) of the fluorescence intensity of the *acdh-1::gfp* expression. Animals were fed the microbial ensemble or monocultures for 72 h, and fluorescence levels were quantified. (C) Distribution of *C. elegans* in bacterial lawns. Percentage of nematodes localized at the center or border of the lawn 72 h post-exposure to different bacterial conditions. (D) Quantification of pharyngeal pumping rates. Pumps per minute were measured in nematodes feeding on the microbial ensemble and bacterial monocultures to assess feeding behavior. (E) DAF-16::GFP localization in response to microbial environments. Percentage of animals with cytosolic or nuclear localization of DAF-16::GFP after feeding on control bacteria (*E. coli* OP50), the microbial ensemble, or *Rhodococcus* at 20°C and following a 37°C heat shock. Same letters denote no statistical differences, while different letters indicate statistically significant differences. Each experiment included three technical triplicates and at least three biological replicates. Complete statistical analyses are detailed in Data set S2.

To test whether the natural microbes support *C. elegans* growth, we fed the ensemble and monocultures to the nematodes and quantified whether animals were adults or L1–L4 larvae and their presence in the lawn at 72 h after hatching. *C. elegans* growth was supported by all bacteria, but *Rhodococcus* and *Chryseobacterium* delayed their development compared to the rest of the strains. This retardation in development was masked in the ensembles with and without amoebae (Fig. 2A). Bacteria that produce vitamin B12 and induce developmental acceleration in *C. elegans* (35) are considered of good quality. The production of vitamin B12 by bacteria can be assessed using animals that express the *acdh-1* promoter driving *gfp* (36), which is turned off in the presence of the metabolite. We fed monocultures and the ensembles to *acdh-1::gfp* nematodes and quantified the fluorescence in the animal’s intestine. *Comamonas*, the ensemble, and *Rhodococcus* turned off the *acdh-1::gfp* expression (Fig. 2B), suggesting they produce vitamin B12.

The location in the lawn was used as a measure of the palatability of the bacteria (37). Nematodes distributed between the center lawn and the border in most bacteria (Fig. 2C). *Rhodococcus*, however, induced half of the population to roam outside the lawn. Differences in development could be attributed to food intake and quality (37). To test the former, we quantify feeding behavior by measuring the pharyngeal pumping rate (pumps per minute) of nematodes after a week of growth in each bacterium. The ensembles were not homogeneous lawns (Fig. 1A), and animals feeding on them showed variability in the pumping rates (Fig. 2D). They stopped for a few seconds in one spot, and then resumed at a higher rate on a different domain of bacteria. Monocultures induced more homogeneous responses. *Comamonas*, *Stenotrophomonas*, and *Chryseobacterium* induced the highest pumping rate, while nematodes on *Rhodococcus* or the ensemble had slower pumping.

A number of animals on monocultures of *Rhodococcus* exhibited the *Dar* (deformed anal region) phenotype previously observed with the *C. elegans* pathogen *Microbacterium nematophilum* (38). To test whether *Rhodococcus* triggered a stress response, we used a strain expressing DAF-16::GFP (39), which localizes to the nucleus upon exposure to starvation, thermic, or pathogenic stress (15, 40). Animals were fed *Rhodococcus*, the ensemble, and the control bacteria *E. coli* OP50. At 20°C, DAF-16 remained cytosolic across all conditions (Fig. 2E), whereas a 37°C heat shock induced nuclear localization in all cases. This result indicates that neither *Rhodococcus* nor the ensemble induces DAF-16-mediated stress responses under our experimental conditions. However, because DAF-16 is not the only transcription factor involved in stress or pathogen responses, we cannot rule out the possibility that *Rhodococcus* may still elicit other stress-related pathways. Additional studies will be needed to fully assess its potential pathogenicity. In summary, *C. elegans* can grow and reproduce in the individual cultures and the ensemble. The observed differences in development, fertility, and pharyngeal pumping rates likely reflect metabolic differences between bacterial species, rather than overt pathogenicity.

### Natural microbial ensembles induce dauer formation

To study the long-term dynamics of the microbial consortia, we co-cultured *C. elegans* with the bacterial community at 20°C and monitored the growth of both the nematode and the individual bacterial species. We quantified bacteria as colony-forming units (CFU), amoebae as individual cysts, and nematodes as total individuals and stage of development every 7 days for 8 weeks. Initial co-cultures contained equal inoculums of each bacterium, a swab of amoebae, and five wild type parental L4 *C. elegans* nematodes on each plate.

**Fig 3:**
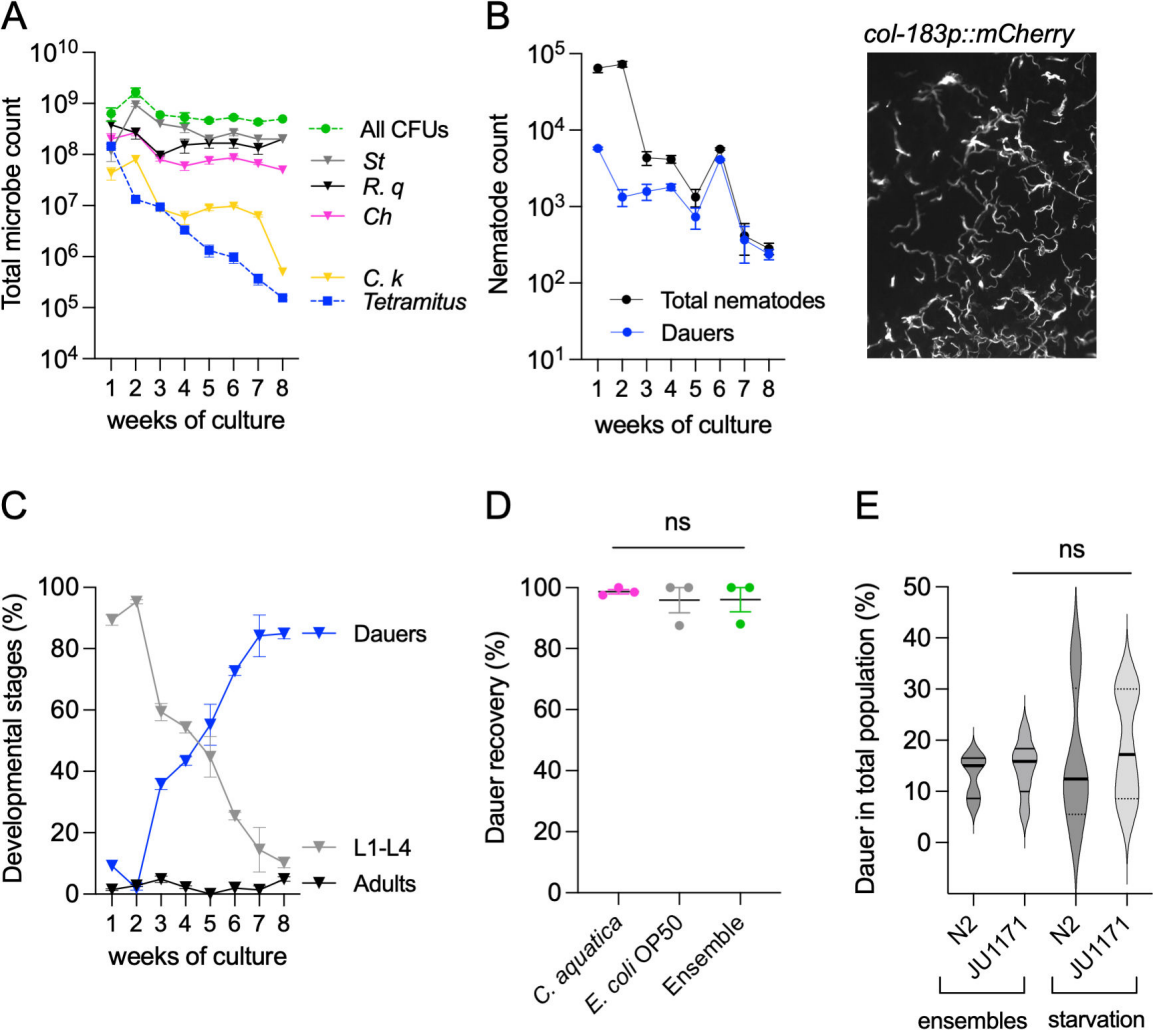
DaFNE and multi-species interactions in a long-term *C. elegans* natural ensemble (A). Total microbial counts of bacteria and amoebae in the ensemble over an 8 week experiment at 20°C. Colony-forming units (CFUs) are shown for total bacteria, individual bacterial species, and *Tetramitus* quantified as individual cysts or trophozoites. (B) Total nematodes and dauer counts in the microbial ensemble over 8 weeks. Inset image shows fluorescent dauer larvae (*col-183p::mCherry*) displayed in monochrome. (C) Percentage composition of the nematode population over 8 weeks categorized into dauers, adults, and non-adult larvae in the microbial ensemble. (D) Recovery from DaFNE. Percentage of dauer larvae that successfully recovered and developed into L4 larvae after 24 h or adults after 48 h when transferred to *E. coli* OP50, *C. aquatica*, or the microbial ensemble. (E) Dauer formation in the wild *C. elegans* isolate JU1171. Dauer induction was quantified after 1 week of exposure to the microbial ensemble or under starvation conditions. *P* value significance levels: 0.1234 (ns). Each experiment included three technical triplicates and at least three biological replicates. Complete statistical analyses are detailed in Data set S2.

Total bacterial CFUs in the ensemble remained stable over 8 weeks (Fig. 3A), although individual bacterial species fluctuated weekly (Fig. 3A and Fig. S3A through D). Among the bacterial species, only *Comamonas* decreased to 10^5^ CFU (Fig. S3A), suggesting that *C. elegans* preferentially feeds on *Comamonas* over other bacteria. *Tetramitus* cysts declined from 10^8^ in the first week to 10^5^ by the 8th week (Fig. 3A) possibly due to predation by other members of the ensemble, competition for resources, or natural attrition over time.

Nematodes reached 60 × 10^3^ individuals the first week and decreased to 300 by the 8th week (Fig. 3B). Unexpectedly, a large number of second-generation (F2) nematodes entered diapause within the first week, despite abundant resources (Fig. 3B), demonstrating that dauers can be induced in the presence of natural microbial ensembles. To quantify dauers, we used a strain that carries the *P_col-183_::dsRed* construct (41), which is expressed only in dauer entry. The inset picture in Fig. 3B shows dauers under the fluorescent stereoscope. While the total number of dauers and other stages decreased steadily toward the 8th week (Fig. 3B; see adults and larvae in Fig. S3E), their proportion in the population increased from 10 to 85% (Fig. 3C). We termed this response dauer formation on naturally derived ensembles (DaFNE).

We next tested whether dauers induced by natural bacteria could exit diapause to colonize other bacteria. We collected 2-week-old dauers from the ensemble with 1% SDS, a treatment that only dauers survive but also induces dauer recovery (42). We placed post-SDS dauers onto *E. coli* OP50, *C. aquatica*, and ensemble lawns and tested their recovery. Consequently, 24 h after, most animals recovered, regardless of the bacteria they were in (Fig. 3D), and by 48 h, all of them were adults showing that DaFNE dauers are viable and capable of colonizing other niches.

To test whether wild *C. elegans* isolates also undergo DaFNE, we exposed JU1171 nematodes isolated from Concepción, Chile, to the bacterial ensembles. We also quantified their dauer entry rate following *E. coli* OP50 exhaustion. JU1171 nematodes formed wild-type levels of dauers under starvation and also exhibited DaFNE at a rate comparable to N2 animals (Fig. 3E), suggesting that DaFNE is a naturally occurring response in diverse *C. elegans* populations.

### DaFNE requires intact microbial communities

**Fig 4:**
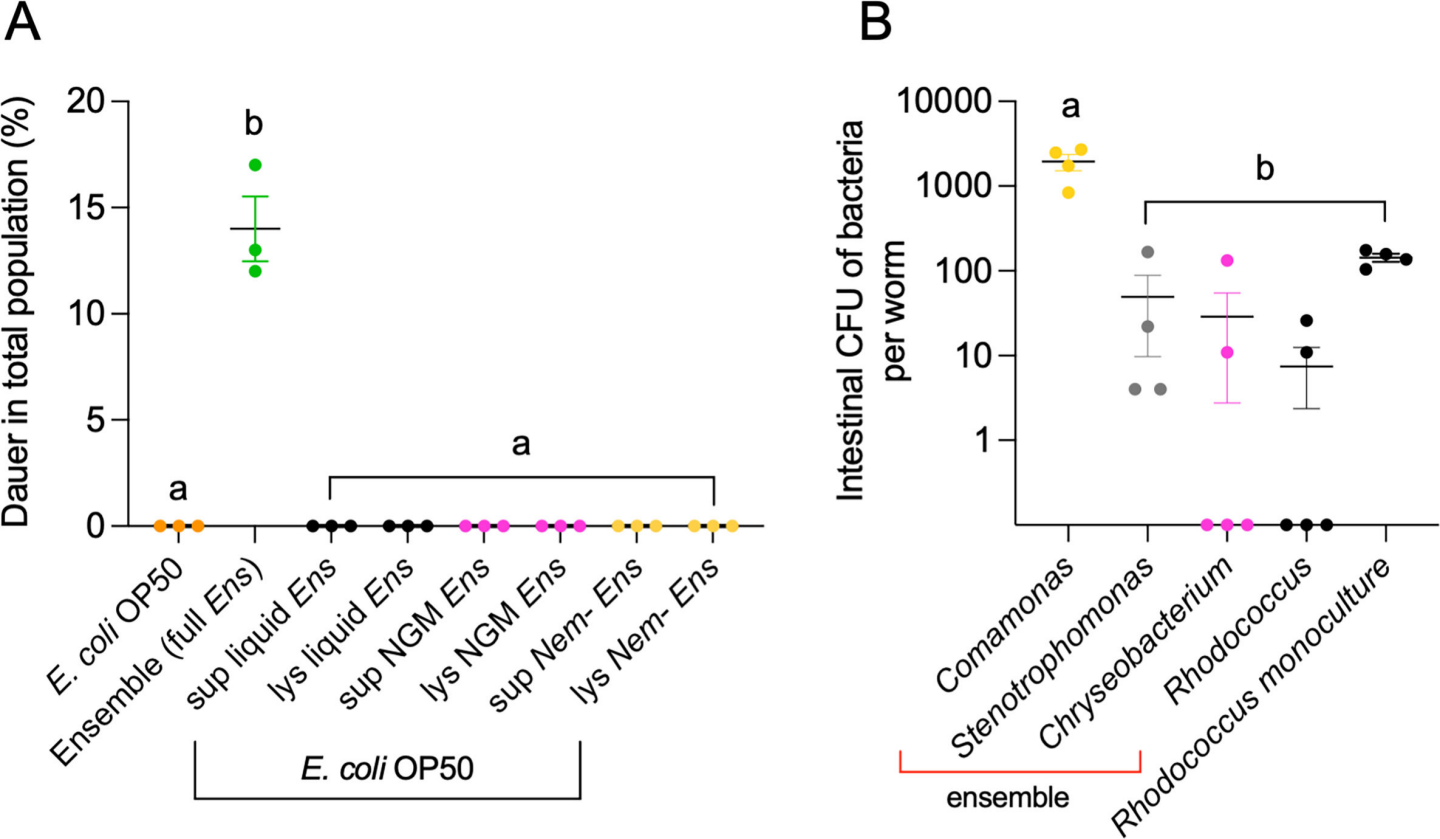
DaFNE induction requires intact microbial communities (A). Dauer formation in response to microbial supernatants and lysates. Nematodes were grown for 1 week on either standard *E. coli* OP50, the full microbial ensemble (*Comamonas*, *Stenotrophomonas*, *Rhodococcus*, and *Chryseobacterium*, full Ens), or *E. coli* OP50 supplemented with ensemble-derived supernatants (Sup) or lysates (Lys). Supernatants and lysates were obtained from (i) the microbial ensemble grown in liquid LB overnight (Liquid Ens), (ii) the microbial ensemble grown on solid NGM plates for 8 days (NGM Ens), and (iii) the microbial ensemble co-cultured with nematodes for 6 days in NGM plates (Nem-Ens). (B) Intestinal bacterial colonization in nematodes exposed to different microbial conditions. Colony-forming unit (CFU) counts of individual bacterial species in the intestines of nematodes feeding on the full microbial ensemble or *Rhodococcus* monocultures. Same letters denote no statistical differences, while different letters indicate statistically significant differences. Each experiment included three technical triplicates and at least three biological replicates. Complete statistical analyses are detailed in Data set S2.

Microbiota effects on host physiology can arise through sustained microbial colonization over multiple generations (15) or via transient exposure to secreted metabolites (7, 43, 44). To test whether DaFNE can be triggered by secreted metabolites—either from bacteria alone or through bacteria–nematode interactions—we examined supernatants and lysates from three different conditions: (i) the microbial ensemble grown overnight in liquid culture, (ii) the ensemble grown on agar NGM plates for 8 days, and (iii) ensembles co-cultured with nematodes for 6 days. These supernatants and lysates were added to standard *E. coli* OP50 cultures, which do not induce dauer formation. Nematodes were then allowed to feed on these supplemented cultures for 1 week. To prevent *E. coli* OP50 depletion and rule out starvation-induced dauer formation, animals were transferred to fresh plates with supplements on the fourth day. Nematodes consuming these supernatants or lysates did not enter dauer (Fig. 4A), indicating that DaFNE requires an intact microbial community rather than secreted metabolites alone.

Because DaFNE requires intact bacterial communities, we next investigated whether the microbial ensemble influences intestinal colonization under dauer-inducing conditions. After 1 week, the predominant intestinal species was *Comamonas* (Fig. 4B) with an average of 3,000 CFUs per nematode, followed by *Stenotrophomonas* and *Chryseobacterium* with 140 and 28 CFU, respectively. *Rhodococcus* was present in very low numbers, averaging only 7 CFUs (Fig. 4B). In contrast, when nematodes were grown on *Rhodococcus* monocultures, they consistently harbored around 10^2^ CFUs per nematode (Fig. 4B). This result indicates that while *Rhodococcus* readily colonizes the *C. elegans* intestine in monoculture, its abundance is restricted in the ensemble, suggesting that microbial interactions modulate bacterial colonization (45).

### Individual bacteria differ in their ability to sustain nematodes and induce DaFNE

**Fig 5:**
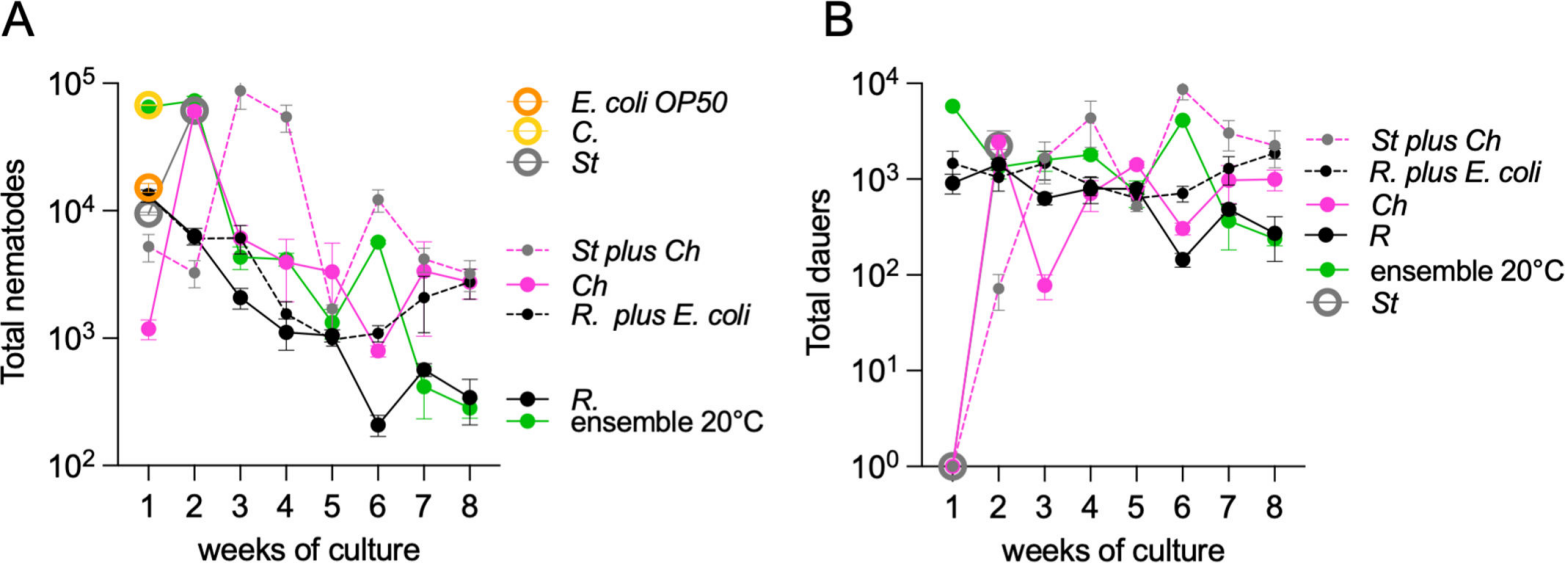
Effects of monocultures and co-culture on nematode growth and DaFNE. (A, B) Population dynamics and dauer formation in different microbial conditions. (A) Total population percentage of nematodes grown for 8 weeks on monocultures of *E. coli* OP50, *Comamonas* (C), *Stenotrophomonas* (St), *Chryseobacterium* (Ch), *Rhodococcus* (R), or bacterial co-cultures and the full microbial ensemble. (B) Percentage of dauers within the total nematode population under the same conditions. Each experiment included three technical triplicates and at least three biological replicates. Complete statistical analyses are detailed in Data set S2.

We examined whether bacterial monocultures could persist for 8 weeks in the presence of *C. elegans*, how their longevity was compared to *E. coli* OP50, and whether they induced diapause. *Comamonas* and *E. coli* OP50 were depleted within a week, resulting in populations of 67,000 and 13,000 non-dauer animals, respectively (Fig. 5A and Fig. S4A). Monocultures of *Stenotrophomonas* lasted only 2 weeks and showed the presence of dauers (Fig. 5B and Fig. S4A). In contrast, *Chryseobacterium* and *Rhodococcus* monocultures persisted beyond 8 weeks (Fig. S4B and C), supporting over 2,000 and 300 total nematodes, respectively, by the end of the experiment (Fig. 5A).

Dauer formation was triggered early in *Rhodococcus* monocultures, whereas *Chryseobacterium* induced dauer entry in the second week and sustained it throughout the experiment (Fig. 5B). The co-culture of *E. coli* OP50 and *Rhodococcus* allowed the former to persist for 8 weeks (Fig. S4D) without reducing the number of dauers (Fig. 5B). A similar effect was observed with *Chryseobacterium* and *Stenotrophomonas* in co-culture, where both species survived for 8 weeks (Fig. S4E), with a dauer formation pattern that differed from either monoculture (Fig. 5B). These findings indicate that bacterial interactions within the ensemble sustain individual species for at least 8 weeks, whereas only *Rhodococcus* and *Chryseobacterium* monocultures can persist in the long term on their own. Additionally, we found that a variety of environmental bacteria induce DaFNE, potentially regulating nematode population growth.

### Nematode life history traits vary between microbial ensembles and monocultures

**Fig 6:**
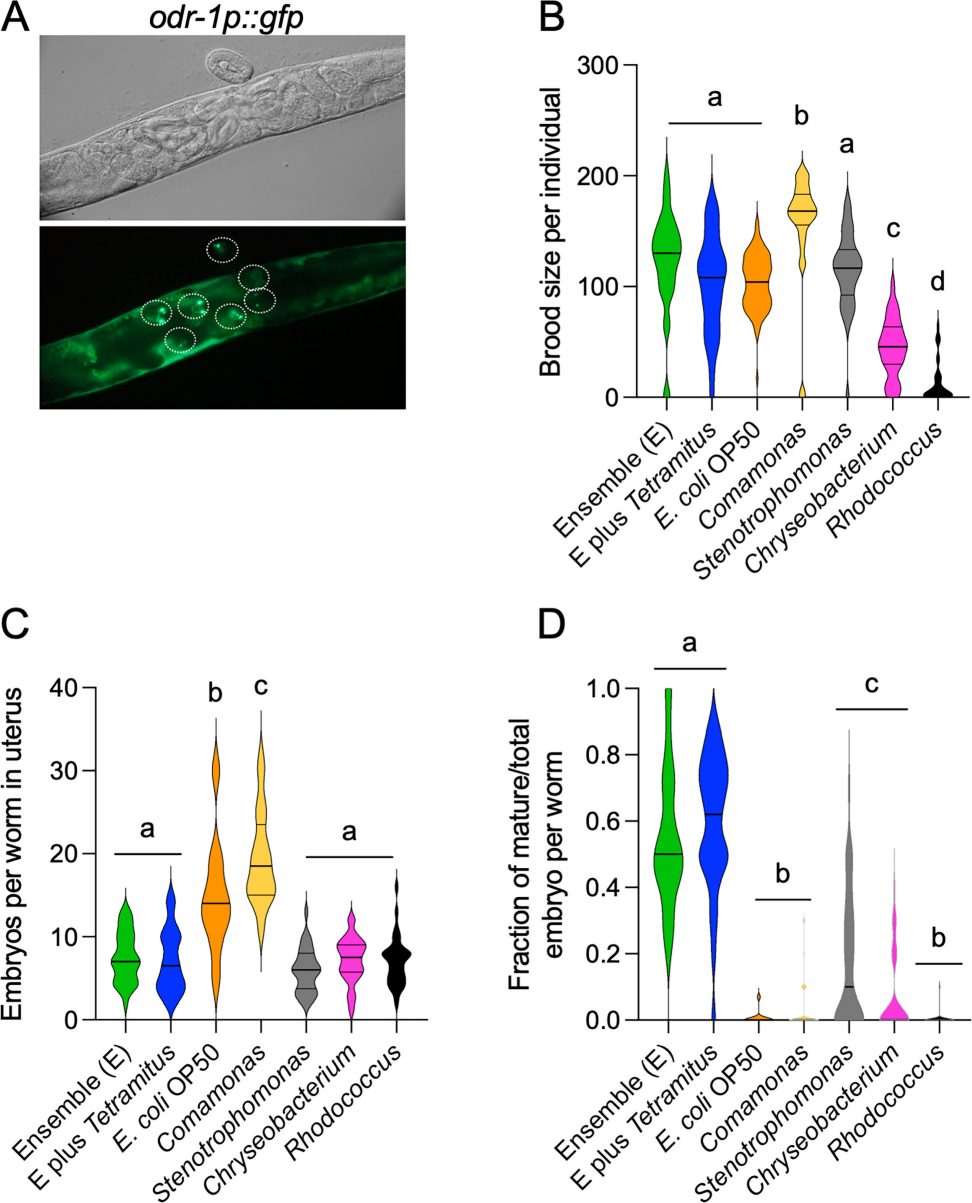
Nematode fertility and embryo maturity in microbial co-cultures (A–D). Effects of microbial co-cultures on nematode reproduction and embryonic development after 1 week. (A) Nomarski (top) and fluorescence (bottom) images of nematodes expressing GFP in the AWB and AWC neurons under the *odr-1* promoter. White circles indicate late-stage embryos identified by their characteristic fluorescence pattern. (B) Number of live progeny laid by individual adults over 72 h. (C) Number of embryos *in utero* after 1 week in the ensemble co-cultures. (D) Proportion of embryos expressing GFP in the AWB and AWC neurons relative to the total number of embryos *in utero*. Same letters denote no statistical differences, while different letters indicate statistically significant differences. Each experiment included three technical triplicates and at least three biological replicates. Complete statistical analyses are detailed in Data set S2.

A reduction in *C. elegans* fertility could serve as a strategy to prevent resource exhaustion. To explore nematode fertility in the context of natural bacteria, we quantified several reproductive parameters: the number of embryos laid by individual nematodes during 3 days since the beginning of adulthood, the average number of embryos *in utero* per animal, and the developmental maturity of embryos in gravid *C. elegans* feeding on monocultures and microbial ensembles for 1 week. To quantify the reproductive output, we tracked the live brood size of individual F2 hermaphrodites for 3 days post-adulthood using a stereoscope and counted *in utero* embryos via imaging under a 40× objective on an upright microscope. To assess embryo maturity, we used transgenic nematodes expressing *GFP* under the *odr-1* promoter in the AWB and AWC neurons, a marker of late embryogenesis (41). We classified GFP-positive embryos as mature (Fig. 6A).

Nematodes cultured in microbial ensembles produced an average of 100 live progenies comparable to those grown on *E. coli* OP50 (Fig. 6B). Although the mean values were similar, data variability was greater in ensembles containing amoebae (Fig. S5A), suggesting that *Tetramitus* may influence the availability of certain bacterial species in the co-cultures. The number of progeny *in utero* was notably lower in the ensembles (Fig. 6C) with a higher proportion of GFP-positive embryos than in *E. coli* or any other monoculture (Fig. 6D). Variability was again greater in cultures with amoebae than in bacterial monocultures (Fig. S5B and C).

The brood size and number of embryos *in utero* for nematodes cultured on *Stenotrophomonas* monocultures were similar to those observed in the ensembles (Fig. 6B). In contrast, other monocultures showed significant differences compared to the consortia (Fig. 6B). For instance, *Comamonas* induced the largest brood size (Fig. 6B), with many embryos *in utero* (Fig. 6C), most of which had not yet expressed *odr-1* GFP (Fig. 6D). On the contrary, despite variability, *Rhodococcus* led to fewer progeny (Fig. 6B), with GFP-negative embryos *in utero* (Fig. 6D). As the consortium forms microdomains (Fig. 1A), nematodes likely receive intermittent contributions from both bacteria and amoebae, shaping their life history traits. The observed variation in embryo retention and maturity aligns with previous findings that *C. elegans* egg-laying behavior is highly plastic and environmentally influenced (46).

### *C. elegans* preys on amoebae but does not contribute to its intestinal microbiota

**Fig 7:**
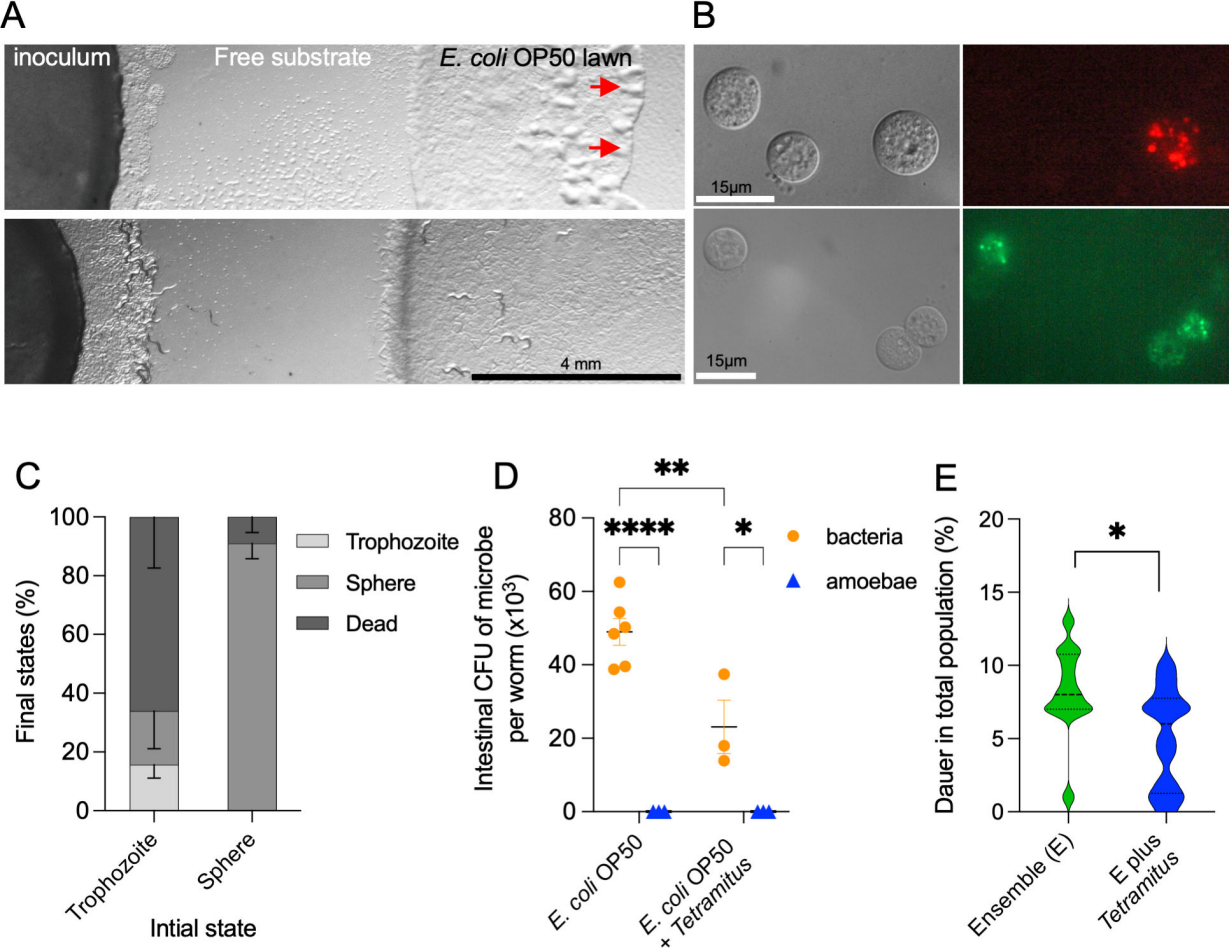
Interactions between *C. elegans* and *Tetramitus* in the microbial ensemble (A). Colonization experiment of the ensemble containing amoebae in *E. coli* OP50-seeded NGM plates. The top panel shows a plate without nematodes, while the bottom panel includes *C. elegans*. The image was taken 3 days after placing synchronized L1 larvae. A visible colonization front (red arrows) in the *E. coli* OP50 lawn is observed only in the plate without nematodes, where amoeba cyst patches and individual *Tetramitus* are present in the free NGM substrate. (B) Cysts of *Tetramitus* after feeding on *E. coli* OP50 labeled with intracellular red and green fluorescent proteins. Note not all cysts contain fluorescent bacteria within their structure. (C) Percentage of *Tetramitus* trophozoites or spheres (initial state) transitioned into trophozoites, remained as spheres, or died after encountering nematodes. Amoebae stages were observed on a microscope pad at 400× magnification. “Sphere” refers to either cysts or newly formed round structures. (D) Colony-forming units (CFU) of intestinal bacteria or amoebae in nematodes fed on either *E. coli* OP50 alone or *E. coli* with *Tetramitus* co-cultures. (E) Dauer percentage in animals feeding on microbial ensembles with or without *Tetramitus*. *P* value 0.1234 (ns), 0.0332 (*), 0.0021 (**), 0.0002 (***), and 0.0001 (****). Each experiment included three technical triplicates and at least three biological replicates. Complete statistical analyses are detailed in Data set S2.

The amoeba *Tetramitus*, a key member of the microbial ensemble, interacts dynamically with *C. elegans*, influencing both feeding behavior and microbial composition. *Tetramitus* thrives on *E. coli* OP50, proliferating rapidly (Fig. 7A, upper panel), with *E. coli* cells remaining viable inside its cysts (Fig. 7B). However, *Tetramitus* growth ceases upon *C. elegans* introduction (Fig. 7B, bottom panel), suggesting that *C. elegans* may prey on the amoebae. To directly observe this interaction, we mounted non-anesthetized nematodes, along with bacteria and amoebae, on a microscope pad and recorded the feeding behavior of *C. elegans* in the presence of motile and non-motile *Tetramitus*. Nematodes readily ingested *Tetramitus* trophozoites but not cysts (Movie S3; Fig. 7C). Upon contact with *C. elegans*, *Tetramitus* frequently adopted a spherical shape, likely as a defensive response to nematode suction (Movie S4; Fig. 7C).

Beyond ingestion, we examined whether amoebae trophozoites establish residency in the nematode intestine or were simply digested. Using a similar protocol for CFU quantification, we plated the intestinal contents on both LB and NGM plates, the latter of which favors amoebae growth. *Tetramitus* failed to grow on either plate after 1 week, suggesting that trophozoites do not survive nematode ingestion (Fig. 7D). Additionally, nematodes grown on bacterial monocultures harbored higher *E. coli* OP50 CFUs than those co-cultured with amoebae, indicating that *Tetramitus* consumption reduced bacterial intake. In summary, these results show that while *C. elegans* can ingest *Tetramitus*, amoebae are not part of its resident microbiota.

Finally, we tested whether amoebae influence DaFNE by comparing dauer formations in microbial ensembles with or without *Tetramitus*. While the overall trend remained unchanged, the presence of *Tetramitus* led to a slight but measurable reduction in dauer formation after 1 week (Fig. 7E).

### Temperature-driven changes in ensemble composition and nematode DaFNE

Temperature, along with other environmental cues, is a key regulator of dauer formation (14). To assess temperature-driven effects on microbial and nematode dynamics, we cultured ensembles at 15, 20, and 25°C following the experimental design of Fig. 3.

**Fig 8:**
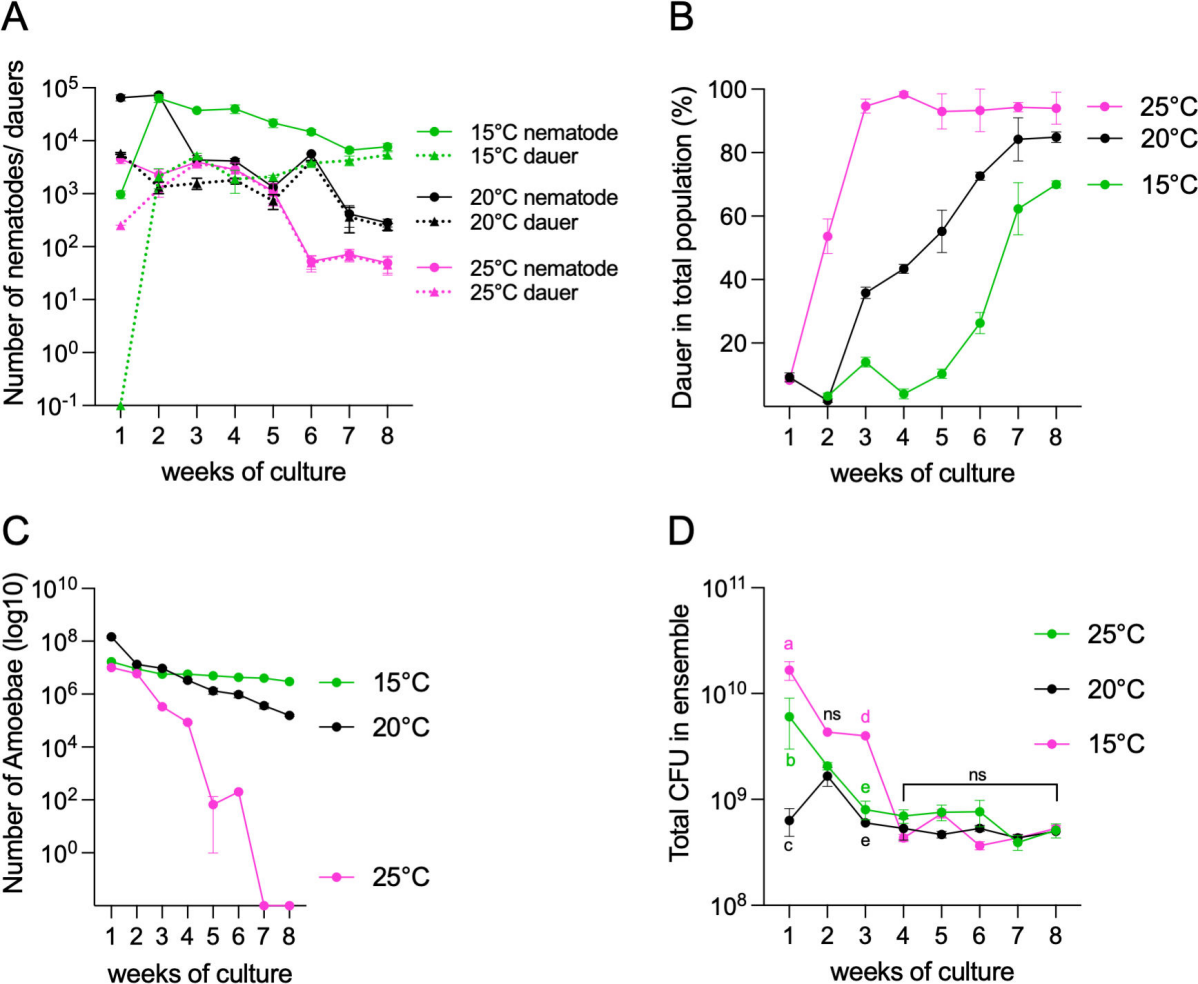
Temperature-driven changes in ensemble composition and dauer formation (A). Total nematodes and dauers at 15, 20, and 25°C over an 8 week period. (B) Percentage of dauers in the total population at each temperature. (C) Number of *Tetramitus* amoeba cysts (log10 scale) tracked over time across different temperatures. (D) Total bacteria colony-forming units (CFUs) calculated as the sum of all bacterial CFUs at each temperature over the 8 week period. Statistical analyses compare each temperature condition at each week. Same letters denote no statistical differences, while different letters indicate statistically significant differences. Each experiment included three technical triplicates and at least three biological replicates. Complete statistical analyses are detailed in Data set S2.

During the first week, nematode numbers were lowest at 15°C (1,000), highest at 20°C (65,000), and intermediate at 25°C (4,000). By the third week, population dynamics reversed, and by the 8th week, the population at 15°C was larger (average 8,000 animals, Fig. 7A and Fig. S4A) compared to only 49 animals at 25°C (Fig. 8A and Fig. S4B). The adult population remained relatively stable at 15°C (Fig. S4E), while it steadily declined at 25°C (Fig. S4F). A similar pattern was observed in young (L1) larvae, with an increase in numbers at 15°C (Fig. S4G) and a significant decrease at 25°C (Fig. S4H). These results indicate that lower temperatures better sustain *C. elegans* populations.

At 25°C, DaFNE began in the first week with ~4,500 animals—nearly 10 times fewer than at 20°C (65,000) or 15°C in the second week (64,000; Fig. 8A and Fig. S4A through D). The percentage of dauers in the total population increased weekly at all temperatures, though the differences between temperatures were significant each week. At 25°C, dauer formation reached 94% by the third week and remained stable until the experiment’s conclusion. At 15 and 20°C, dauer percentages rose gradually to 70 and 84%, respectively (Fig. 8B). While DaFNE increased with rising temperatures, there were notable differences, with a high penetrance at 25°C. This suggests that in natural environments subject to thermal stress, the survival of the population may rely heavily on dauers. We next examined the growth of amoebae and bacteria at the different temperatures. At 15°C, amoebae remained stable at ~3 × 10^6^, whereas at 20°C, numbers declined to 10^5^. At 25°C, *Tetramitus* numbers steadily declined and were exhausted by the 7th week (Fig. 8C), indicating that amoebae are not viable at sustained higher temperatures.

Bacteria were initially more abundant at 15°C than at 20 or 25°C during the 1st and 3rd weeks, but by the 4th week, total CFU counts were similar across all temperatures (Fig. 8D). Individually, the four bacterial species fluctuated during the first 2–3 weeks at 15°C (Fig. S4I and L). At 25°C, only *Comamonas* and *Chryseobacterium* exhibited fluctuations in the 2nd week (Fig. S4I and K), while *Stenotrophomonas* and *Rhodococcus* remained stable. While the bacterial component of the ensemble displayed small changes in quorum across the three tested temperatures, amoebae and nematodes experienced more drastic perturbations in growth and dauer formation.

### DaFNE is a multigenerational response to natural bacteria

**Fig 9:**
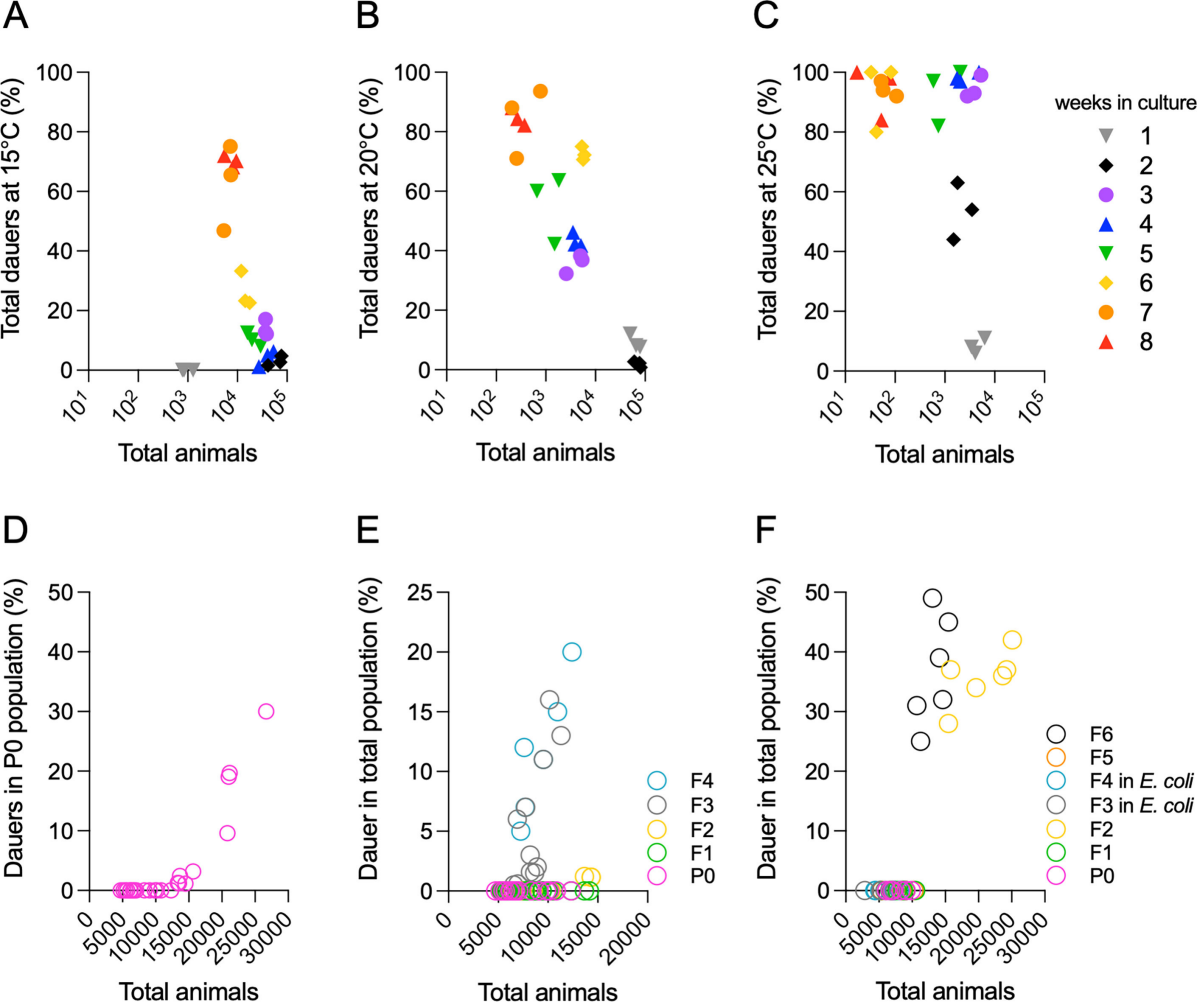
Generational effects on DaFNE in a long-term paradigm. (A–C) Percentage of dauers at 15°C (A), 20°C (B), and 25°C (C) over successive generations in a long-term paradigm, where neither bacteria nor animals were replaced between weeks. (D) Percentage of dauers in the P0 generation as a function of the increasing total population size. (E) Percentage of dauers in an intergenerational paradigm where each generation was interrupted by hypochlorite treatment before re-exposure to the ensemble. (F) Percentage of dauers in a transgenerational paradigm, in which nematodes experienced two generations in *E. coli* before reintroduction to the ensemble. Each experiment included three technical triplicates and at least three biological replicates. Complete statistical analyses are detailed in Data set S2.

Crowding and food limitation are well-established triggers of *C. elegans* diapause (12). Despite continuous food availability in the 8 week paradigm, dauer formation progressively increased across generations (Fig. 9A through C), while total nematode numbers declined at all tested temperatures (Fig. S6A, B and 3B). To determine the population threshold for DaFNE induction, we collected P0 embryos from *E. coli* OP50-fed hermaphrodites and exposed varying population densities in the bacterial consortium. After 3 days in the ensemble, we counted the total population and the number of dauers. Dauer formation did not occur below 13,000 animals. Between 13,000 and 15,000 naïve animals were needed for 1% dauer formation, while populations exceeding 20,000 induced 10% (Fig. 9D).

To quantify the cumulative effect of DaFNE across generations, we extracted each generation using hypochlorite treatment and reintroduced the embryos to freshly seeded ensemble plates. Intergenerational effects build up when the stimulus is continuously present, whereas transgenerational effects persist after at least two generations without stimulus exposure (47). To evaluate intergenerational effects, we initiated the experiment with P0 animals and quantified dauer formation in F1–F4 descendants. In each generation, we used fewer than 15,000 embryos (below the DaFNE threshold for P0 and F1). The F2 generation required approximately 15,000 nematodes to induce more than 1% dauers, similar to P0s, while F3 and F4 showed higher dauer formation with fewer nematodes, supporting an intergenerational effect of DaFNE (Fig. 9E).

Transgenerational effects manifest as either increased dauer formation (higher percentage) or accelerated onset (one generation earlier) after re-exposure to the ensembles following a two-generation break on *E. coli* OP50. While the magnitude of DaFNE was similar between F6 and F2, after re-exposure, dauers formed one generation sooner (Fig. 9F), occurring in F6 instead of F7. Additionally, fewer animals were required to induce dauer formation in F6 compared to P0 animals (Fig. 9D). These findings demonstrate that DaFNE has both inter- and transgenerational effects.

### Nematode pheromone synthesis is needed for DaFNE

**Fig 10:**
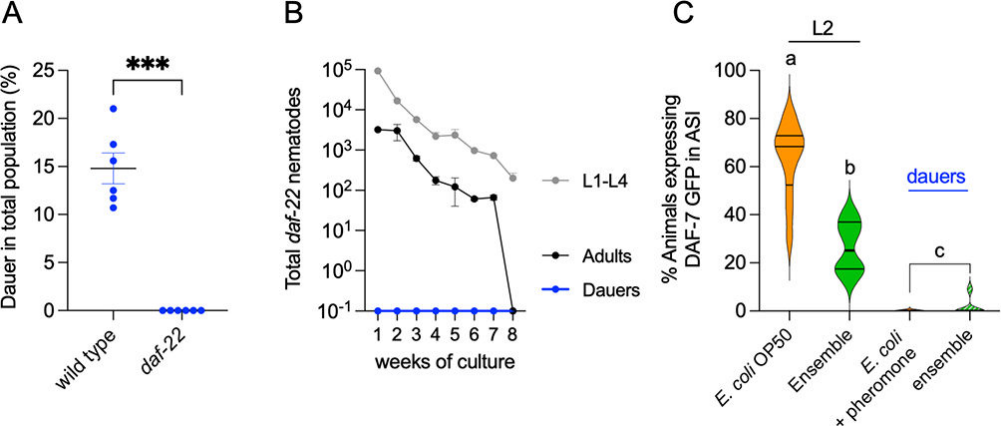
Pheromone pathway mutations disrupt DaFNE (A). Percentage of dauer formation in wild-type and *daf-22* mutants after 1 week in the microbial ensemble. (B) Total nematodes by developmental stage (dauer, larvae, and adult) in wild-type and *daf-*22 mutants over an 8-week long-term paradigm. (C) Percentage of animals expressing GFP in the ASI neuron under the *daf-7* promoter across different bacterial food conditions and developmental stages. *P* value 0.1234 (ns), 0.0332 (*), 0.0021 (**), 0.0002 (***), and 0.0001 (****). Same letters denote no statistical differences, while different letters indicate statistically significant differences. Each experiment included three technical triplicates and at least three biological replicates. Complete statistical analyses are detailed in Data set S2.

In *C. elegans*, pheromones mediate social interactions through specific receptor pathways (48, 49). To determine whether DaFNE depends on pheromone synthesis, we used *daf-22* mutants, which lack a key enzyme for ascaroside production (48). Although *daf-22* mutants can enter diapause at high temperatures (14), they fail to do so in response to other stressors (15, 48, 50). After 1 week, *daf-22* mutants failed to form dauers (Fig. 10A). Even after 8 weeks of continuous exposure to the ensemble (Fig. 10B), no dauer formation was observed, demonstrating that pheromone production is required for DaFNE.

In ASI neurons, pheromones suppress the *daf-7/*TGF-β expression, triggering dauer entry. Conversely, food availability restores *daf-7* transcription and promotes normal development (51, 52). We measured the GFP expression driven by the *daf-7* promoter in ASI neurons of both developing animals and dauers within the natural ensemble. Under normal feeding conditions on *E. coli* OP50, 63% of L2 animals displayed a high *daf-7* expression in ASI neurons, whereas most pheromone-induced dauers lacked this expression (Fig. 10C). Within the ensemble, only 26% of animals retained a high *daf-7* expression, significantly fewer than those fed *E. coli* OP50 (Fig. 10C). The majority of ensemble-induced dauers showed low *daf-7* expression, resembling pheromone-induced dauers (Fig. 10C). This suggests that while DaFNE shares some commonalities with pheromone effects, it is not entirely identical.

### Intestinal dsRNA transporter SID-2 and argonaute RDE-1 are needed for DaFNE

Bacteria–nematode interactions frequently involve the host’s RNA interference (RNAi) machinery and small RNAs from both bacteria and host (6, 53, 54). Mutations in *sid-1* or *sid-2* disrupt RNAi initiated by bacterial dsRNA in the intestine, rendering the response ineffective (25, 26, 55). Once inside the cell, exogenous dsRNAs are processed by the Argonaute protein RDE-1 (27, 28). To test whether DaFNE requires cell-autonomous and systemic RNAi, we exposed *sid-1* (*pk3321*), *sid-2* (*qt42*), and *rde-1* (*ne219*) mutants to the microbial ensembles and measured population growth and dauer formation after 1 week. To confirm that these mutants retained the ability to enter diapause, we starved them and measured dauer formation.

**Fig 11:**
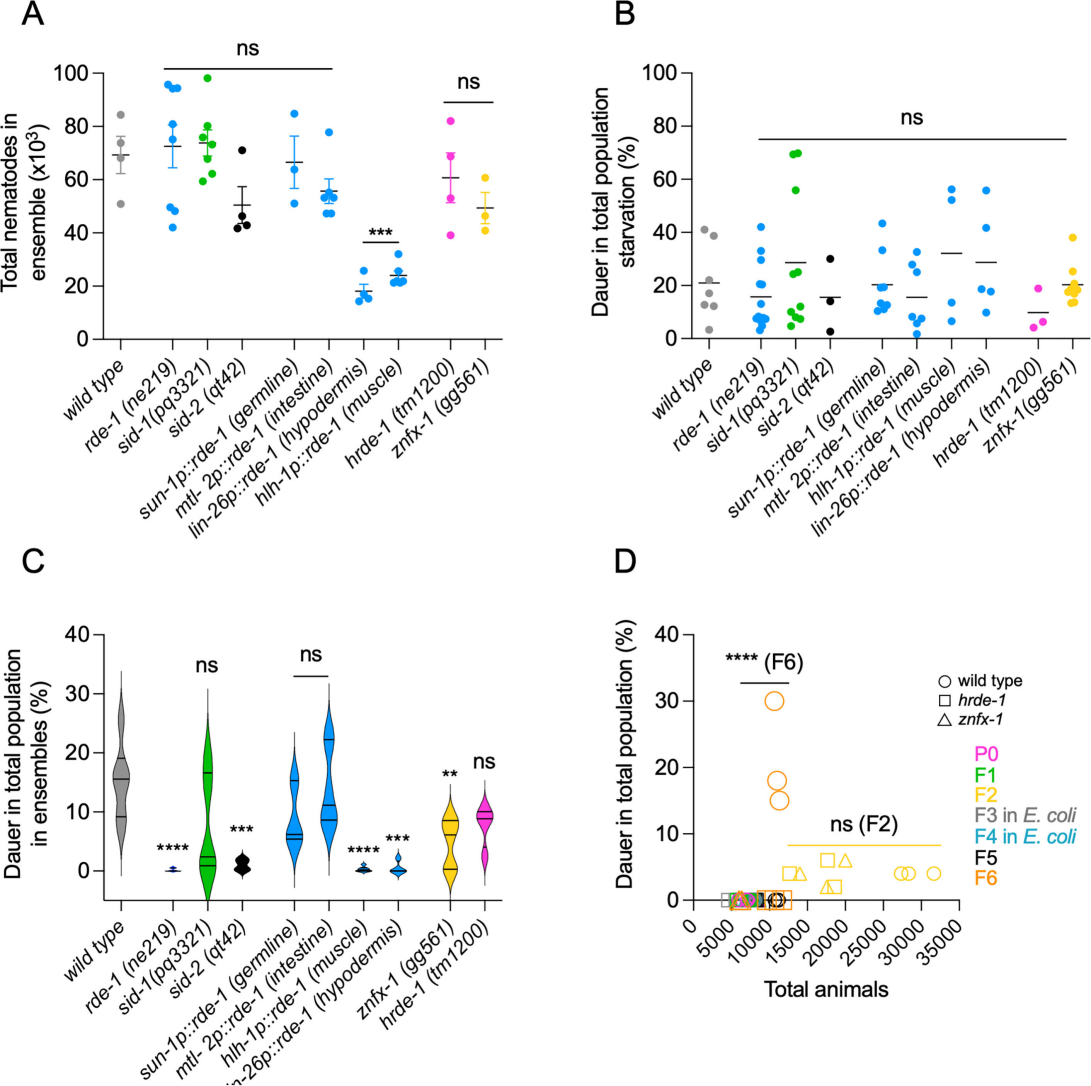
The RNAi machinery is needed for DaFNE initiation and inheritance. (A) Total nematode population size in wild-type, *rde-1*, *sid-1*, *sid-2*, *znfx-1*, and *hrde-1* mutants, as well as *rde-1* rescue strains expressing *rde-1* in the germline (*sun-1p::rde-1*), intestine (*mtl-2p::rde-1*), hypodermis (*lin-26p::rde-1*), and muscle (*hlh-1p::rde-1*). (B) Percentage of dauer formation in wild-type and mutant strains under starvation conditions. (C) Percentage of dauer formation in the microbial ensemble for wild-type and RNAi-related mutants. (D) Dauer formation in a transgenerational paradigm for wild-type, *rde-1*, and *znfx-1* animals. Comparisons shown are between F2 and F6 generations for wild-type, *hrde-1*, and *znfx-1* animals (ns for F2 and **** for F6). *P* value 0.1234 (ns), 0.0332 (*), 0.0021 (**), 0.0002 (***), and 0.0001 (****). Each experiment included three technical triplicates and at least three biological replicates. Complete statistical analyses are detailed in Data set S2.

*sid-1*, *sid-2*, and *rde-1* mutants showed wild-type population numbers (Fig. 11A) and formed normal numbers of dauers upon starvation (Fig. 11B). However, DaFNE was impaired in *sid-2* and *rde-1* mutants (Fig. 11C), suggesting that dsRNA uptake by intestinal cells via the transporter SID-2, as well as the processing of primary small interfering RNAs, are required for DaFNE. The *sid-1* mutant displayed variable DAFNE levels, but its response was not significantly different from wild-type animals (average 8.85, SEM 3.7), suggesting that other systemic RNAi components may compensate for *sid-1* loss (56, 57).

To explore the tissue-specific role of *rde-1* in DaFNE, we used strains with *rde-1* rescued specifically in the germline, intestine, muscle, and hypodermis. Rescue in the intestine and germline fully restored wild-type growth rates and DaFNE formation, while rescue in muscle and hypodermis resulted in populations approximately one-third the size of wild-type animals (Fig. 11A) and a complete failure to form DaFNE. Given their reduced growth, we cannot determine whether the DaFNE defect in these strains is due to insufficient quorum or because *rde-1* rescue in these tissues is irrelevant to DaFNE. Importantly, all rescue strains formed normal numbers of dauers under starvation (Fig. 11B), indicating that their ability to enter diapause was not broadly compromised.

We next examined whether effectors of heritable RNAi contribute to the initiation or maintenance of DaFNE across generations. Specifically, we tested the nuclear Argonaute HRDE-1 (29) and the helicase ZNFX-1 (58), both of which are required for heritable RNAi. *hrde-1* mutants exhibited wild-type responses during the initial establishment of DaFNE (Fig. 11C), whereas *znfx-1* mutants showed variable responses that were significantly different from wild type. Both mutants formed normal levels of dauers under starvation (Fig. 11C).

To determine whether RDE-1 and ZNFX-1 are necessary for transgenerational DaFNE, we performed experiments analogous to those in Fig. 9F. Animals were exposed to bacterial ensembles (P0-F2) transferred to *E. coli* OP50 for two generations (F3–F4), and then re-exposed to the ensembles (F5–F6). At each stage, we quantified total population size and dauer formation. As expected, wild type, *hrde-1*, and *znfx-1* mutants all formed dauers in the F2 generation, with total populations ranging from 12,000 to 31,000 (Fig. 11D). However, as observed previously (Fig. 9F), neither wild-type nor mutant strains formed dauers in the F5 generation. By F6, only wild-type animals exhibited robust dauer formation at significantly higher rates than in F2 (Fig. 11D). In contrast, both *hrde-1* and *znfx-1* mutants failed to enter dauer in the F6, unlike the wild-type nematodes (Fig. 11D), suggesting that the RNAi machinery is involved not only in the initiation of DaFNE but also in its epigenetic memory across generations. In summary, our findings indicate that bacterial nematode interactions rely on RNA-based communication, culminating in a heritable epigenetic effect that governs dauer formation across multiple generations.

## DISCUSSION

We developed a framework for the long-term cultivation of an ensemble consisting of bacteria, amoebae, and nematodes to study their growth dynamics and nematode behavior in this setting. Unlike standard 60 mm NGM plates seeded with *E. coli* OP50 or *Comamonas aquatica*, which typically exhaust within a week when starting with 30–50 embryos, ensemble conditions supported the sustained presence of reproductive nematodes and abundant bacteria for at least 1 week. Notably, nematodes entered diapause despite the availability of resources and the absence of apparent stress, a phenomenon we termed Dauer Formation on Naturally derived Ensembles or DaFNE.

### DaFNE differs from starvation-induced dauer formation

A central question raised by our findings is whether DaFNE is merely a starvation response or a distinct ecological adaptation. Unlike dauer formation triggered by food depletion, DaFNE occurs in the presence of abundant bacterial resources, challenging the assumption that it results from nutritional stress. Moreover, while starvation-induced dauer formation does not require the RNAi machinery, DaFNE is dependent on RDE-1, a primary Argonaute required for exogenous RNAi (27, 28). Furthermore, DaFNE displays transgenerational inheritance, with dauer formation accelerating upon re-exposure to the ensemble after a two-generation pause—an effect that requires HRDE-1 and ZNFX-1, known effectors of heritable RNAi (29, 58). This suggests that DaFNE is an adaptive response to microbial interactions rather than a consequence of poor nutrition.

### Microbial interactions and the role of pheromones in DaFNE

The repression of DAF-7/TGF-β expression in the ASI neurons during DaFNE and the *daf-22* requirement are reminiscent of a pheromone-induced dauer formation (52). However, unlike stress-induced diapause in response to pathogens or adverse conditions (15, 20, 54), DaFNE is triggered by a natural microbial community that does not induce classical stress responses. This raises the intriguing possibility that soil bacteria regulate nematode population dynamics by promoting dauer formation as part of a cooperative ecological strategy (59). In this context, DaFNE may allow nematodes to conserve resources, even when food is available or to prepare for sudden environmental changes, such as temperature fluctuations or pathogen threats. Indeed, our results show that higher temperatures lower the population density required for DaFNE and accelerate its onset, reinforcing its potential ecological significance (60–62).

### Amoebae may indirectly modulate the microbial landscape

Our results indicate that amoebae do not directly contribute to DaFNE but may still play a role in shaping the microbial environment. *C. elegans* readily ingest amoebae, such as *Tetramitus*, but these organisms do not become part of its intestinal microbiota. However, *Tetramitus* may influence nematode behavior by altering bacterial viability or growth within the ensemble, creating dynamic microenvironments that modulate nematode life history traits. Such interactions highlight the complexity of the nematode–bacteria–amoebae holobiont (63) and suggest that amoebae may contribute indirectly to nematode population regulation by modifying bacterial composition.

### RNAi effectors in DaFNE regulation

Our findings suggest that DaFNE is mediated by a combination of microbial metabolites and nematode pheromones. Given the dependence of DaFNE on the RNAi machinery, bacterial small RNAs and host RNAi pathways may act in concert to regulate dauer formation. Additionally, bacterially derived chemicals are known to modulate host behavior (7–10) and complex traits, such as animal quiescence in natural systems, likely involve multiple molecular pathways within the holobiont (64, 65). For example, *Comamonas*, a consistent component of the nematode intestine, produces vitamin B12, which is associated with developmental acceleration in *C. elegans* (35), whereas *Rhodococcus* delays development and reduces progeny, which may explain its low prevalence in the intestinal microbiota.

In summary, bacteria, nematodes, and amoebae interact to create a long-term, self-sustained niche where pheromones, microbial metabolites, and the RNAi machinery regulate animal quiescence. DaFNE represents a novel adaptation to natural microbial communities, with potential implications for understanding interspecies communication, population regulation, and nematode ecological strategies. Future studies will focus on identifying the molecular cues driving DaFNE and their interactions with host signaling pathways, exploring the long-term tradeoffs and fitness benefits of DaFNE across generations (46, 66).

## MATERIALS AND METHODS

### Collection and culture of natural microbial ensembles

Microbial ensembles were collected from naturally occurring moss in the soil of an urban area in Santiago, Chile (−33.432480912271856, –70.6147650371416) during summer. A piece of moss approximately 27 cm^3^ was collected, placed in a sterile container, and transported to the laboratory. The moss was then placed in a petri dish containing sterile distilled water for 1–2 h. After this incubation period, motile nematodes, rotifers, and protozoans became visible under a dissection microscope. A small sample of the liquid containing soil debris was spread on LB plates to allow bacterial growth. Another sample containing nematodes and soil was placed on an *E. coli* OP50-seeded agar plate using a worm pick.

The bacterial inoculum containing *Tetramitus* was incubated at room temperature (RT) for several days. Wild nematodes did not survive, but *Tetramitus* spread rapidly over the *E. coli* OP50 lawn, replacing the entire lawn with cysts 48–72 h after inoculation. Meanwhile, an orange-colored bacterial ensemble grew more slowly and steadily; this difference in growth rate allowed for easy separation of amoebae and bacteria. Room temperature fluctuated between approximately 15 and 25°C over a 24 h period.

Individual bacterial strains were isolated by repeated streaking until single colonies were obtained.

### Maintenance and growth of bacteria

Bacteria were grown overnight on Luria Bertani (LB) plates at RT (natural ensembles) and 37°C (*E. coli* OP50) from glycerol stocks. The next morning, a scoop of the inoculum was cultivated in liquid LB at 200 rpm at RT for 48 h for monocultures and the ensemble and 37°C for 6 h for *E. coli* OP50. Subsequently, 150 µL of the bacterial culture was seeded onto 60 mm NGM (2% agar plates, 0.25% tryptone [2.5 g/L], 0.3% sodium chloride [3.0 g/L], 1.5% agar, 1 mM calcium chloride, 1 mM magnesium sulfate, 25 mM potassium phosphate, pH 6.0, and 5 µg/mL cholesterol) plates and allowed to dry overnight before use.

### Maintenance and growth of *C. elegans*

Wild-type, transgenic, and mutant *C. elegans* were maintained at 20°C, as reported previously (67). The following nematode strains were used: wild type (N2); PS8438 [*syIs600* (*col-183p*::mCherry + *odr-1p*::GFP)]; NL3321 [*sid-1*(*pk3321*)]; WM27 [*rde-1* (*ne219*)], DCL569 {mkcSi13 [sun-1p::rde-1::sun-1 3′UTR + unc-119(+)]}, NR222 [kzIs9 (lin-26p::rde-1 + rol-6(su1006)], [rde-1 (ne219)], NR350 [kzIs20 [hlh-1p::rde-1 + sur-5p::NLS::GFP], [rde-1 (ne219)]; IG1839 [frSi17 [mtl-2p::rde-1 3′UTR], frIs7 [nlp-29p::GFP + col-12p::DsRed], rde-1(ne300)]; YY538 [*hrde-1*(*tm1200*)]; YY996 [*znfx-1*(*gg561*)]; TJ356 [*zIs356* [*daf-16p::daf-16a/b*::GFP + *rol-6*(*su1006*)]; DR476 [*daf-22* (*m130*)]; FK181 [*ksIs2* [*daf-7p*::GFP + *rol-6*(*su1006*)]; and the *C. elegans* wild isolate, JU1171. All animals were maintained in NGM plates seeded with *E. coli* OP50 at 20°C before using or feeding with other bacteria.

### Maintenance and growth of *Tetramitus*

*Tetramitus* plates were maintained in NGM seeded with *E. coli* OP50 bacteria. A total of 100–1,000 cysts were taken from a fully colonized plate using a platinum loop that was 1–2 mm in diameter. Viable cysts can be obtained after months of storage at RT in sterile conditions. We used 1 to 7-day-old fully colonized plates for cyst inoculation.

### DNA extraction and the identification of bacteria and amoebae in the ensemble

To identify bacteria, we first exhausted the cultures in LB plates until isolates were obtained. Individual bacteria on LB plates were sent to Macrogen for sequencing. PCR of 16S rRNA genes was performed using 27F and 1492R primers, and sequencing was conducted using 785F and 907R primers, which were the inter-primers, to identify bacteria.

### Genus identification and phylogenetic trees of bacterial 16S RNA sequence samples

For each sample, its 16S RNA sequence was used as a query for National Center for Biotechnology Information (NCBI)’s BLASTN (68) (default megablast option [69] using NCBI’s nucleotide (nt) database [70, 71]), followed by the download of the sequences. A biological sampling of surrounding species at different taxonomic levels was performed to broaden the phylogenetic background of the query species, allowing its location more clearly in the bacteria tree. This was done by displaying in a phylogenetic tree all 16S RNA sequences from the species of the same genus as the samples that resulted from BLASTN, together with additional 16S RNA sequences from each of the taxonomic levels of the genus, in each case. The taxonomy levels of each sample were taken from the Taxonomy Database from NCBI (70). The 16S RNA sequences were downloaded from the NCBI’s Nucleotide Database.

The maximum likelihood (ML) tree is searched from a starting tree made from a multiple sequence alignment build using MAFFT (72). As most of the downloaded 16S rRNA sequences are described as “partial sequences,” we used MAFFT’s most accurate algorithm, L-INS-I, using the “localpaired” option with 1,000 refinement iterations and the default fasta format output. The result was input to iqtree2 (73) for model inference (option -m TESTONLY), followed by several runs of iqtree2 for the ML tree space search. Each iqtree2 run specified the best evolutionary model (option -m); the number of bootstrap iterations, a thousand, (-b 1,000); a perturbation option (-perturb) (we ran three searches for each following perturbation value: 0.3, 0.5, 0.7, and 0.9); and the option --nstop 500, which is the number of additional tries the program does when an impossible tree is reached. Moreover, the -abayes option for Bayesian bootstrap was used. A total of 12 simultaneous tree space searches per sample were performed using up to 48 central processing unit threads, option -t AUTO.

### Genomic DNA extraction and the identification of amoebae

Genomic DNA from amoebae was extracted using the Quick-DNA Miniprep Plus Kit (Zymo Research). Specifically, we used the protocol for Biological Fluids and cells with the following modifications: initially, the sample was incubated with 200 µL BioFluid & Cell Buffer with 20 µL of Proteinase K. Instead of incubating at 55°C for 10 min, we incubated overnight. For whole-genome sequencing, we sent 920 total nanograms of gDNA of amoebae eluted in elution buffer from the Quick-DNA Miniprep Plus Kit. Library construction and sequencing were done by Macrogen using the Truseq Nano DNA Library in a NovaSeq 6000 Sequencer 150 paired-end (150 × 2 bp) 10 Gb/sample.

### Phylogenetic inference amoebae

We started by listing *Tetramitus* relative species taken from previous inferred trees found in the literature (33, 74) and used the internal transcribed spacer or ITS from the 5.8S ribosomal RNA sequence (75, 76) and the ribosomal RNA 18S partial sequence. Both have been used for *Tetramitus* phylogenetic inference (77, 78). We had to compromise to a smaller number of species that had both sequences available instead of the number of species that had only one of the two rRNA sequences.

Thus, we selected from NCBI’s Nucleotide Database (70, 71) (http://www.ncbi.nlm.nih.gov/nucleotide/) the records of the longest ribosomal 18S and 5.8 rRNAs, including the ITS1 and ITS2 sequences found, respectively, in the following species: *Allovahlkampfia* sp. (JQ271670.1 from PKD 2011b type strain PV66; LC106131.1); *Naegleria clarki* (JQ271691.1 from strain 2HZ; AJ566625.1 from type strain BG6); *Paravahlkampfia* sp. (GU230754.1 from A1PW2; AJ698857.2 from isolate 6/3Ab/1B); *Tetramitus dokdoensis* (KY463322.1; KY463323.1); *Tetramitus thermacidophilus* (AJ621575.1; AM950228.1 ecotype Pzc6); *Tetramitus thorntoni* (KT257696.1 from type strain SkGuaT; AJ698843.1 from type strain CCAP1588/8T); *Tetramitus jugosus* (KT257697.1 from type strain PrGuaT; AJ698844.1 from type strain CCAP1559/1T); *Tetramitus entericus* (KC164219.1 from clone CF1-11; AJ698856.1 from isolate C101); and *Vahlkampfia avara* (JQ271723.1 from type strain 4171L; AJ698837.1 from type strain CCAP1588/1AT).

The phylogenetic tree search was performed with the same methodology described above in “Genus identification and phylogenetic trees of bacterial 16S RNA sequence samples.”

### Extended result identification of *Tetramitus*

From the 10 *Tetramitus* classified species, only five counted with their 18S and the 5.8 rRNA, including ITS1 and ITS2 sequences. The evolutionary model inferred as the best model according to the Bayesian information criterion was TIM3 + F + I + G4. The final trees were generated from 789 informative positions from the MSA.

### Eight-week experiments

#### Initial culture

Individual ensemble bacteria were revived from glycerol stocks stored at −80°C by plating on LB solid plates (90 mm) and incubating overnight at room temperature (RT; 18–25°C). Amoebae were maintained on NGM plates seeded with *E. coli* OP50 at RT. The following morning, a scoop of each monoculture and a scoop of amoebae were inoculated into 15 mL of liquid LB and shaken at 200 rpm for 48 h at RT. From this culture, 150 µL was seeded onto 60 mm NGM plates and allowed to dry and grow for 48 h to enable amoebae growth within the ensemble. The next day, five L4 nematodes of strain PS8438 were placed onto the 60 mm NGM plates (24 individual plates per experiment) and allowed to grow either at 15, 20, or 25°C for 8 weeks.

#### Quantification of ensemble

Every week, three plates per condition were examined to quantify each species within the ensemble. The entire plates' contents were washed with 1 mL of M9 buffer, collected into a 1.5 mL Eppendorf tube, and centrifuged at 376 ×*g* for 2 min. The supernatant was transferred to a clean Eppendorf tube for bacterial quantification, while the pellet was used for nematode and amoeba quantification.

#### Bacteria

Serial dilutions of the supernatant were prepared from 1:10 to 1:10^7^. A 150 µL aliquot of each dilution was plated on solid LB plates in triplicate. Bacterial counts expressed as CFU were recorded 72 h after inoculation.

#### Amoebae

Amoeba cysts were counted from both the nematode pellet and the bacteria-containing supernatant. Total amoebae counts were the sum of both measurements. Serial dilutions from 1:10 to 10^3^ were prepared, and three separate counts were taken to quantify the amoeba cysts in each sample.

#### Nematodes

The nematode pellet was resuspended in 500 µL of M9. Dilutions of 10 µL or 1:10 were used to count: (i) total nematodes, (ii) dauer larvae, (iii) adults, and (iv) L1–L4 larvae. PS8438 dauers were counted under a fluorescence dissecting microscope. An average of three measurements per condition was recorded for each count.

### Supplementation of *E. coli* with ensemble supernatant and lysate

To test dauer formation after 1 week, we collected supernatants and lysates from three different conditions for *E. coli* OP50 supplementation: (i) 10 mL LB liquid cultures of the microbial ensemble grown overnight, (ii) ensembles grown on 60 mm NGM plates for 8 days, and (iii) ensembles co-cultured with nematodes for 6 days on 60 mm NGM plates.

For liquid cultures, samples were centrifuged using a Beckman Coulter Allegra 21R Centrifuge with a Beckman S4180 Swing Bucket Rotor at 5,450 ×*g* for 10 min to separate the bacterial pellet from the supernatant. The supernatant was filtered twice using syringe-driven filters (30 mm, 0.2 µm; Biofilter). A 150 µL aliquot of the filtrate was added to NGM plates seeded with *E. coli* OP50. To prepare the lysate, 1 mL of lysis buffer from the DNeasy PowerSoil Pro Kit (Qiagen) was added to the pellet, incubated for 30 min at RT, and centrifuged again at 5,450 ×*g* for 10 min. A 150 µL aliquot of the resulting lysate supernatant was then added to NGM plates seeded with *E. coli* OP50.

For ensembles grown on solid media and ensembles co-cultured with nematodes, the contents of each 60 mm NGM plate were harvested in 1.5 mL of M9 buffer by pipetting until all bacteria (or bacteria and nematodes) were fully collected. The suspension was transferred to Eppendorf tubes and centrifuged at 9,331 ×*g* for 10 min at 4°C in a 5424R Eppendorf Centrifuge. The supernatant was separated from the pellet, transferred to a new Eppendorf tube, and filtered twice with syringe-driven filters (30 mm, 0.2 µm; Biofilter). A 150 µL aliquot of this filtrate was added to NGM plates seeded with *E. coli* OP50.

To prepare lysates from the pellets obtained in this step, 1 mL of lysis buffer from the DNeasy PowerSoil Pro Kit (Qiagen) was added to each pellet, incubated for 30 min at RT, and then centrifuged at 9,331 ×*g* for 10 min in a 5424R Eppendorf Centrifuge. A 150 µL aliquot of each lysate supernatant was subsequently added to NGM plates seeded with *E. coli* OP50.

### Quantification of dauer larvae

#### Using a fluorescent marker

The entire nematode population on each plate was collected in 1 mL of M9 medium. A 10 µL aliquot of a 1:10 dilution was counted three times to determine the total population and the number of dauers using a Nikon SMZ1270 fluorescence dissection microscope. Animals exhibiting clear dauer morphology and expressing *mCherry* in the cuticle were classified as dauers.

#### Using 1% SDS

Wild-type or mutant animals were washed off the plates in 1 mL of M9, and the resulting pellet was resuspended in 500 µL of M9. To count the total population, a 1:10 dilution was prepared in M9. For dauer counting, a separate 1:10 dilution was prepared in 1% SDS (10 µL in 90 µL of 1% SDS). Animals were incubated in the 1% SDS solution for 15 min. A 10 µL sample from each dilution was used to count live dauers and the total population. The percentage of dauers in the total population was then plotted.

### Quantification of multigenerational effects of DaFNE

#### Intergenerational paradigm

PS8438 embryos obtained from hypochlorite treatment were deposited onto plates, and the resulting embryos were counted and placed on plates with the ensembles. The number of embryos deposited varied depending on the quorum range evaluated (5,000 to 30,000 embryos). Seventy-two hours after hypochlorite treatment, the total number of nematodes and dauers was counted. Each plate was washed with 1 mL of M9 buffer, and the wash was collected in a 1.5 mL Eppendorf tube. The samples were then centrifuged at 376 ×g for 2 min; the supernatant was discarded; and the nematode pellet was resuspended in 1 mL of M9. A 10 µL aliquot of the nematode solution was placed on a slide, and the total number of nematodes and the number of dauers were counted using a Nikon SMZ1270 fluorescence dissection microscope. Dauers were identified as animals expressing the *col-183::mCherry* marker.

After counting, the nematode pellet tubes were centrifuged again; the supernatant was discarded; and 500 µL of hypochlorite solution was added to initiate the hypochlorite protocol, which was repeated as described above through the F4 generation. Each new generation was obtained through hypochlorite treatment, and the embryos were placed on newly seeded plates.

#### Transgenerational paradigm

Bacteria and nematodes were grown and treated identically to the intergenerational protocol, with one key difference: F2 embryos were transferred to *E. coli* OP50 plates for 3 days (F3 generation in *E. coli*), and F3 embryos were also transferred to *E. coli* OP50 plates (F4 generation in *E. coli*). Embryos from the F4 generation were then re-exposed to the ensembles (F5 generation), and their embryos were passed to the ensembles for an additional generation.

### Dauer recovery

Dauer animals were separated from non-dauer nematodes using a 1% SDS treatment. The resulting pellets of dauers were placed on seeded plates, with the dauers deposited in a drop outside the bacterial lawn. Dauer exit was assessed at 24 h by counting the number of L4 or non-dauer animals. At 48 h, the number of adults was recorded. Additionally, plates were examined under a fluorescence dissecting microscope to confirm the presence of dauers by the expression of the *col-183p::mCherry* marker.

### Pharyngeal pumping

For each bacterial lawn or ensemble, 10 well-fed adult *C. elegans* were used to quantify the number of pharyngeal pumps per minute. Each pump was manually counted using a counter, with timing controlled by a 1 min timer. Observations were conducted at 10× magnification using a Nikon SMZ745 dissecting microscope.

### Microscopy, photography, and video

Microphotography of *Tetramitus*, *C. elegans*, and natural bacterial cultures was performed at low magnification directly on NGM/agar plates using a Nikon SMZ 745T trinocular dissection microscope with an additional 2× front lens, coupled with a 10× tube lens, and a Sony XCD-SX910 digital camera connected via firewire to a personal computer. Micro-assemblies were also imaged at high magnification using DIC optics on either a Nikon Eclipse Ni or Ti microscope equipped with Canon EOS Rebel T3i and Nikon D780 digital cameras, respectively. Samples were prepared on 2% (wt/vol) agarose pads on a 2 mm glass slide, with a 0.17 mm coverslip placed on top.

Photograph of PS8438 dauers expressing cuticular *mCherry* was imaged directly on their growing plate using a Nikon SMZ 1270 fluorescence dissection microscope at 40× magnification, with an appropriate filter cube for GFP imaging. The Alvium 1800 U-500 Complementary Metal Oxide Semiconductor (CMOS) Sensor (Allied Vision) was set to an exposure time of 220 ms and a gain of 24.1 dB.

### Pheromone extraction

A pheromone mixture was obtained following the method described by Schroeder and Flatt (79), with some modifications. A 1 L culture of PS8438 nematodes initiated from six 10 cm plates containing starved animals was grown for 1 month at room temperature (18–25°C), with agitation at 110 rpm. Experimental NGM plates were prepared with a concentration of 5% (vol/vol) pheromone and seeded with 2% (wt/vol) heat-killed *E. coli* OP50 bacteria 24 h prior to experiments.

Fifteen FK181 adult hermaphrodites were manually transferred to the pheromone–NGM plates using a pick and allowed to lay eggs for 3 h before being removed. The plates were then incubated at 20°C for 72 h. Dauer larvae were manually selected based on morphology and scored. Additionally, 50–100 L2 larvae were grown from a bleach treatment on NGM plates seeded with live *E. coli* OP50, prepared 24 h before incubation, and maintained at 20°C for 24 h.

### Tracking of amoebae and behavioral analysis

Hundreds of *Tetramitus* cysts were transferred from a fully colonized plate onto the NGM substrate placed 1–2 mm away from the edge of the bacterial lawn. Photos were captured every 30 s at 40× magnification using image acquisition tools and custom scripts in MATLAB (MathWorks). Time-lapse activity was assessed by subtracting consecutive images and calculating the absolute value of the intensity change for each pixel. The total number of pixels, where intensity change exceeded the defined threshold, was counted and reported as activity over time. The threshold was determined based on inactive regions of the image at the initial stages of the experiment.

An ad-hoc model was used to fit the cumulative time-lapse activity (CA) described by equation 1:


(1) $$CA\left(t\right)=cIA\left(1-e^{-\left(t-tx\right)/tp}\right)+\frac{cCA}{1+e^{-\left(t-tc\right)/tg}}.$$

In this model, cIA and cCA represent the amplitudes of the inoculum and colonization phases of behavior, respectively. tx relates to excystation kinetics defined by the quiescence observed before a sudden increase in activity. tp represents the natural decay time in the exploration activity of moving trophozoites after excystation. tg (grow time) is the inverse of the natural grow rate in the logistic curve describing the colonization phase. tc (colonization time) is the time at which the logistic grow phase reaches half of its plateau.

### Nematode brood size

Total brood size was counted for 30 randomly selected individual F2 adults grown for 1 week on bacterial natural ensembles at 20°C. The number of live progenies for each adult were counted at RT for three consecutive days. For *E. coli* OP50, the number of individual adults used was 90.

### Quantification of GFP expression in the ASI neurons

The strain FK181 {*ksIs2* [*daf-7p*::GFP + *rol-6*(*su1006*)]} was used to quantify GFP expression in the ASI neurons. Two levels of GFP expression were observed: weak fluorescence limited to the soma without neurite expression and strong fluorescence extending throughout the entire cell, including both the soma and the neurite. Only the strong fluorescence was considered GFP-positive in the analysis. For both *E. coli* and ensemble conditions, dauers were isolated using a 1% SDS treatment and confirmed by morphology. L2 animals were selected based on morphological criteria.

### Quantification of GFP intensity in the intestine

VL749 {*wwIs24* [*acdh-1p::GFP + unc-119*(+)]} nematodes expressing intestinal GFP were imaged directly on their growing plate using a Nikon SMZ 1270 fluorescence dissection microscope at 40× magnification and equipped with an appropriate filter cube for GFP imaging. The Alvium 1800 U-500 CMOS sensor (Allied Vision) was set to an exposure time of 220 ms with a gain of 24.1 dB. Images containing worms were analyzed in ImageJ by selecting the areas corresponding to individual animals and measuring the average pixel intensity.

### Bacterial count (CFU) in nematode intestine

After 7 days in the ensembles, 30 L4 worms of each condition were transferred to a tube with 1 mL of M9. The tubes were centrifuged at 376 ×*g* for 2 min, and the supernatant was removed, leaving approximately 0.1 mL in the tube. The pellet was washed six times with 1 mL of M9 supplemented with 25 mM levamisole (Lev + M9). A 10 µL aliquot of the supernatant was used to seed an LB plate as a control (Control 1).

The pellet was washed three times with 1 mL Lev + M9 containing a mix of antibiotics (50 µg/mL carbenicillin, 25 µg/mL tetracycline, 25 µg/mL chloramphenicol, and 100 µg/mL gentamicin). The tube was incubated with Lev + M9 + antibiotics for 1 h with agitation, with the solution being replaced with a fresh one midway through the hour. After incubation, the pellet was washed with 1 mL of Lev + M9 and centrifuged. A 10 µL aliquot of the supernatant was plated on LB as an additional control (Control 2).

The pellet was washed three more times with 1 mL of Lev + M9, and a final 10 µL aliquot of the supernatant was plated on an LB plate for a third control (Control 3). For colony quantification, 150 µL of the same supernatant was spread on an LB plate until exhaustion.

The worm pellet was then lysed with a pestle for 2 min or until completely dissolved, and the remaining steps followed the original protocol (80). Bacterial colonies were identified based on macroscopic morphological criteria: *Comamonas* appears as small, white, clear, round colonies with a surrounding halo and liquid texture; *Stenotrophomonas* colonies are larger than *Comamonas*, round, with a greenish color and glossy, viscous texture. *Chryseobacterium* colonies are larger than both *Comamonas* and *Stenotrophomonas*, round, with a bright yellow-orange color and an extremely viscous consistency; and *Rhodococcus* colonies are the smallest, round, and dark, with a pale pink matte surface and a non-homogeneous texture.

### Biological and technical replicates and statistical evaluation

Each experiment was conducted in three technical triplicates and included at least three biological replicates. Detailed data for all experiments are provided in Data set S1. Biological replicates are defined as experiments conducted on different days, each containing triplicates of each condition, while technical replicates are defined as triplicates of the same condition performed on the same day. Each figure includes data from at least three experiments (biological replicates) conducted as described. Biological replicates were performed at intervals ranging from 1 day to 1 week.

Statistical evaluation was performed using one- or two-way analysis of variance with post hoc tests, and Student’s *t*-test was used when indicated. All statistical analyses are detailed in Data set S2.

## SUPPLEMENTAL MATERIAL

Data set 1 msystems.01566-24-s0001.xlsx All data.

Data set 2 msystems.01566-24-s0002.xlsx All statistical analyses.

Supplemental figures msystems.01566-24-s0003.pdf Figure S1–S6.

Combined legends for Movies and Datasets msystems.01566-24-s0004.txt Legends for the supplemental movies and data sets.

Movie S1 msystems.01566-24-s0005.mp4 Amoeba and culturable bacteria.

Movie S2 msystems.01566-24-s0006.mp4 Amoebae moving on bacterial lawn.

Movie S3 msystems.01566-24-s0007.mp4 *C. elegans* feeding on trophozoites.

Movie S4 msystems.01566-24-s0008.mp4 Amoeba becoming round.

ASM does not own the copyrights to Supplemental Material that may be linked to, or accessed through, an article. The authors have granted ASM a non-exclusive, world-wide license to publish the Supplemental Material files. Please contact the corresponding author directly for reuse.
