# Supplementary material for: Interspecies relationships of natural amoebae and bacteria with C. elegans create environments propitious for multigenerational diapause
Source: mSystems. 2025 Mar 20;10(4):e01566-24. doi: 10.1128/msystems.01566-24 (PMC12013276; doi:10.1128/msystems.01566-24)
Supplement: Supplemental figures — Figure S1–S6. [file msystems.01566-24-s0003.pdf]

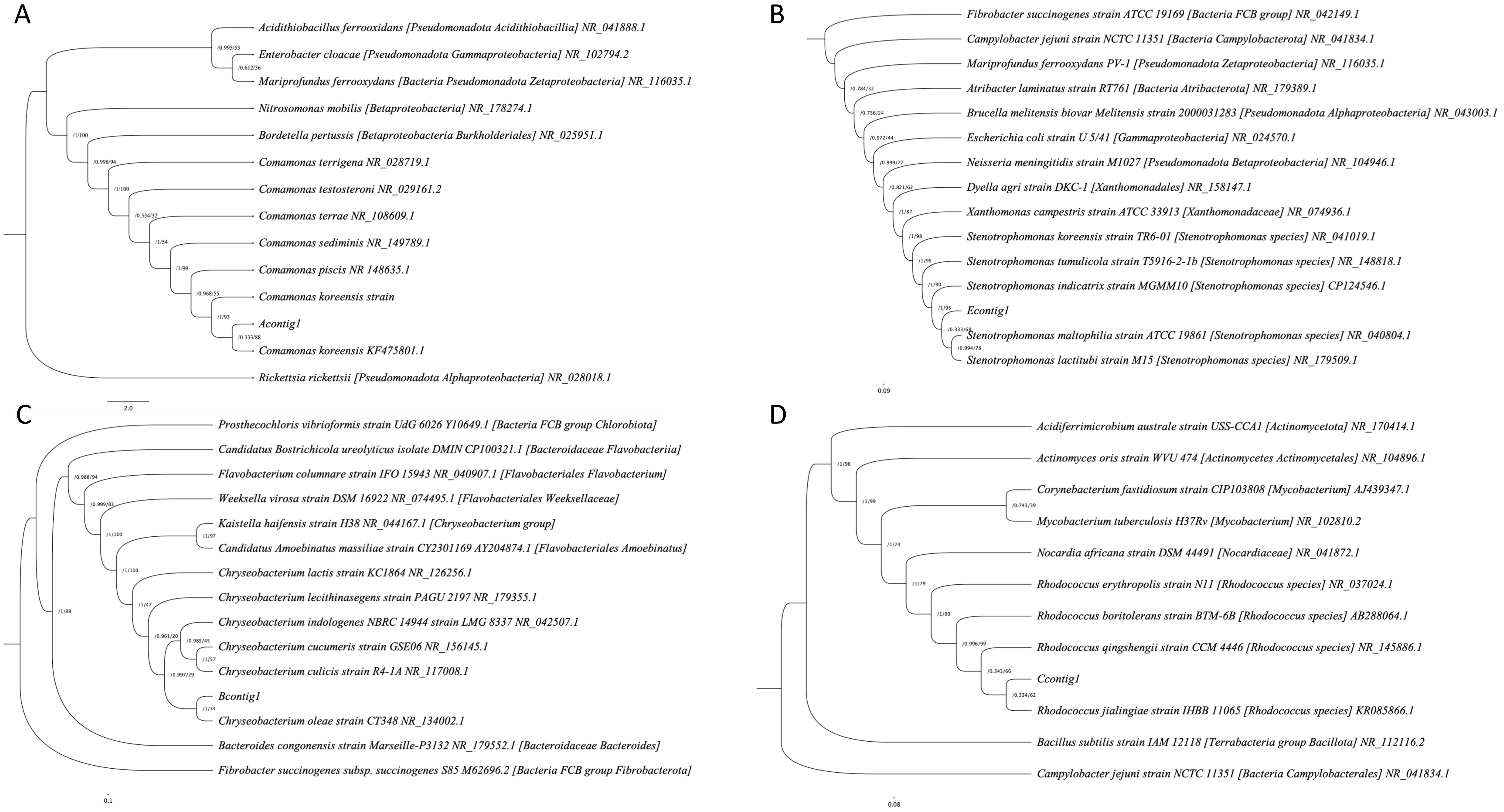

Figure S1. A-D Maximum Likelihood Phylogenetic tree of A-D contigs and surrounding taxonomic representative species. Bootstrap and Bayesian bootstrap values are displayed in the nodes.

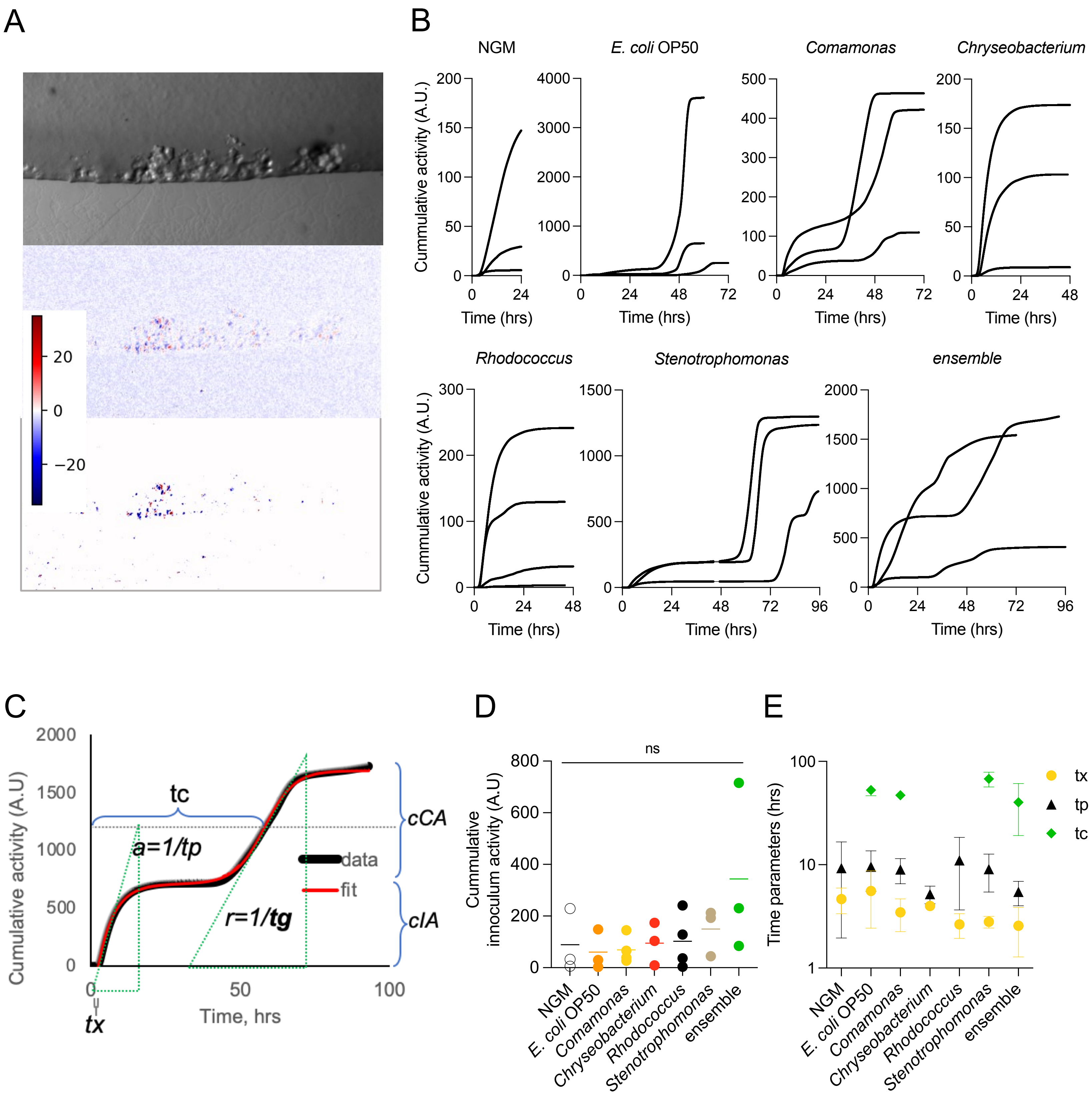

Figure S2. A. *E. coli* OP50-seeded NGM plate being colonized by *Tetramitus*. Top: a single picture of an *E. coli* OP50-seeded NGM plate where the bacterial lawn appears in the upper part of the photograph. Cysts were placed right below the bottom of the picture. After excystation, amoebae traveled the lower half to reach the bacterial lawn boundary, where they settle down and multiply (lumps near the agar/lawn interface). Note the trails left by moving amoebae connecting the bottom with the equator of the picture. Middle: pixel to pixel subtraction of two consecutive pictures taken 0.5 minutes apart. Bottom: pixel differences with absolute value smaller than 8 A.U. were discarded Activity due to amoebae moving around appear as intense blue and red spots. Intensity color scale is on the right (arbitrary units). Bigger patches of activity coincide with lumps in bacterial lawn near the center. **B.** Cumulative activity obtained from time lapse experiments performed on different bacterial lawns. NGM condition contains no bacterial lawn. **C.** *Tetramitus* colonization curve (black thick line) is an example of growth in *Stenotrophomonas* done by plotting the cumulative time-lapse activity over time. Red line is the model fit (see methods) that yields six parameters for each curve: tx, tp, tc, tg, cIA and cCA. **D.** Cummulative inoculum activity amplitude (cIA) for different bacteria. **E.** Excystation time (tx), Exploration time (tp) and colonization time (tc) obtained from parametric fit to equation 1. Experiments that do not support colonization (NGM, *Rhodococcus* and *Chryseobacterium*) have a colonization time tending to infinity (>>100 hours). A.U is arbitrary units. P value 0.1234 (ns). Complete statistical analyses are detailed in **Dataset 2**.

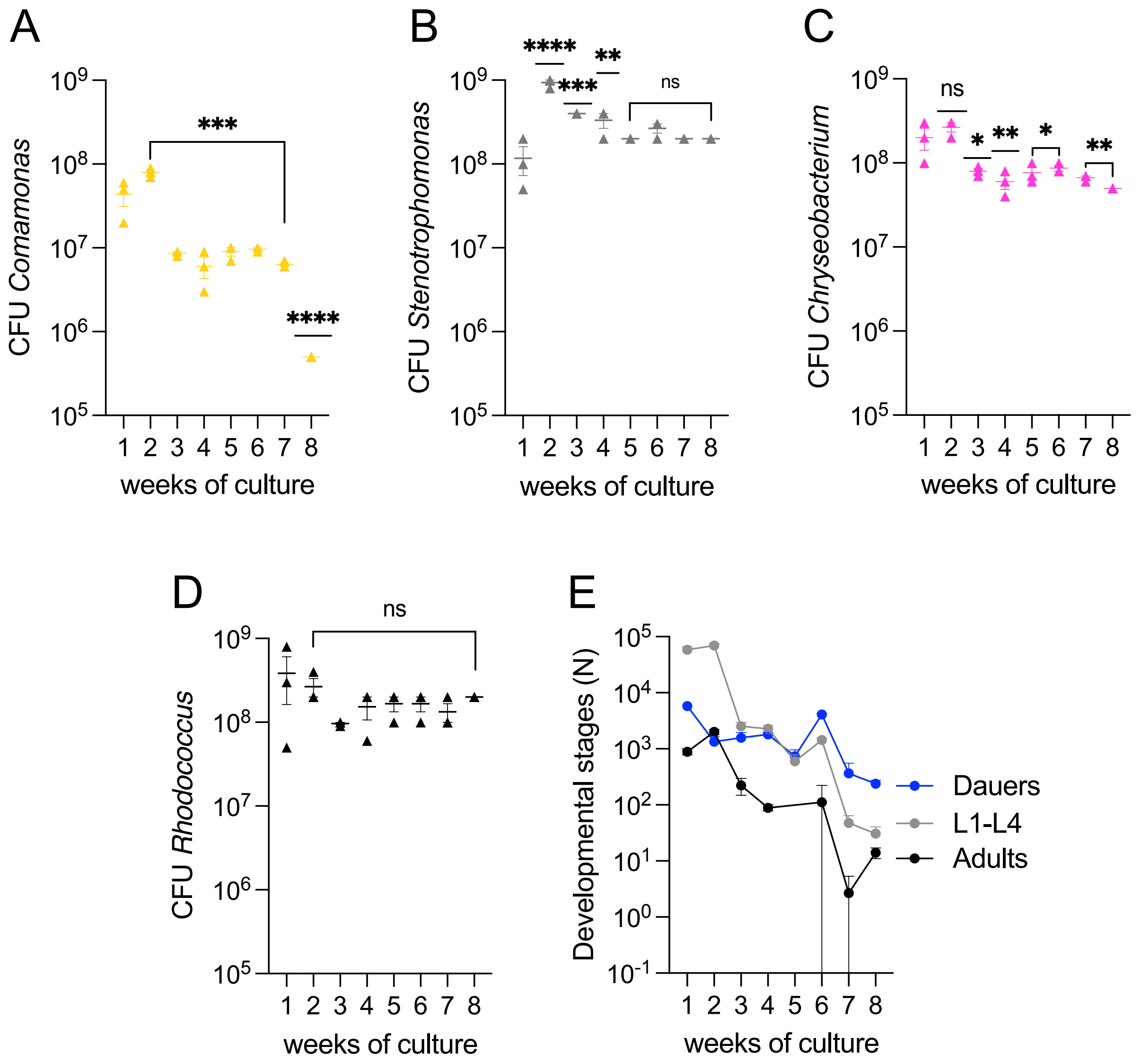

Figure S3. **A-D**. Number of individual CFUs per bacterial species in the ensemble (**A**) *Comamonas* (**B**) *Stenotrophomonas*, (**C**) *Chryseobacterium* and (**D**) *Rhodococcus* during the long-lasting co-culture. **E**. Progression of the numbers of adults, dauers and larvae in the ensemble throughout 8 weeks. P value 0.1234 (ns); 0.0332 (*); 0.0021 (**); 0.0002 (***); 0.0001 (****). Each experiment included three technical triplicates and at least three biological replicates. Complete statistical analyses are detailed in Dataset 2.

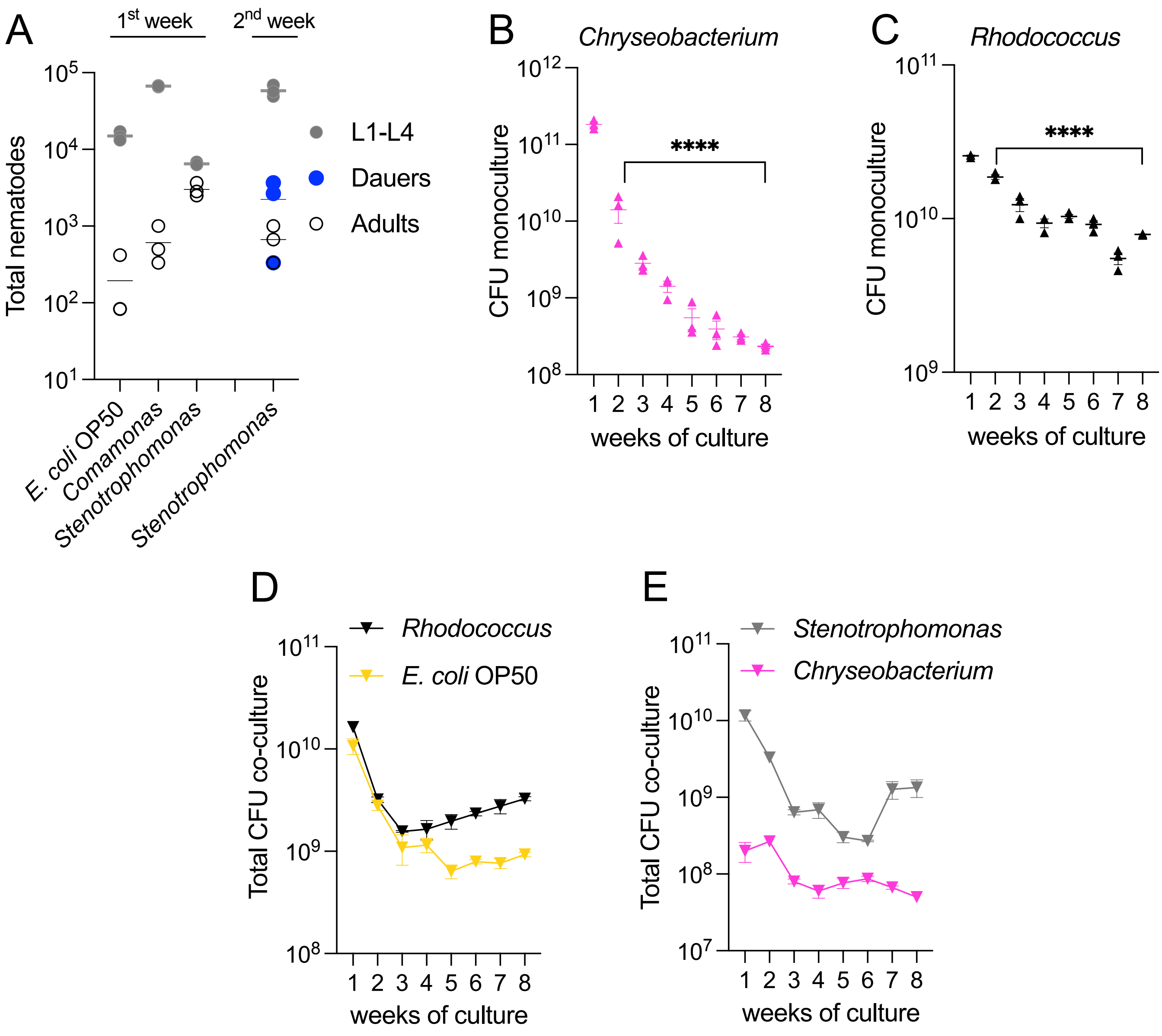

Figure S4. A. Total nematodes as adults, dauers and larvae in monocultures of the control strain *E. coli* OP50 and *Comamonas* during the first week of growth and two weeks on *Stenotrophomonas*. **B-E**. CFUs of *Chryseobacterium* (**B**) and *Rhodococcus* (**C**) monocultures and *Rhodococcus* with *E. coli* OP50 (**D**) and *Stenotrophomonas* with *Chryseobacterium* (**E**) co-cultures during eight weeks growing with *C. elegans*. P value 0.1234 (ns); 0.0332 (*); 0.0021 (**); 0.0002 (***); 0.0001 (****). Complete statistical analyses are detailed in **Dataset 2**.

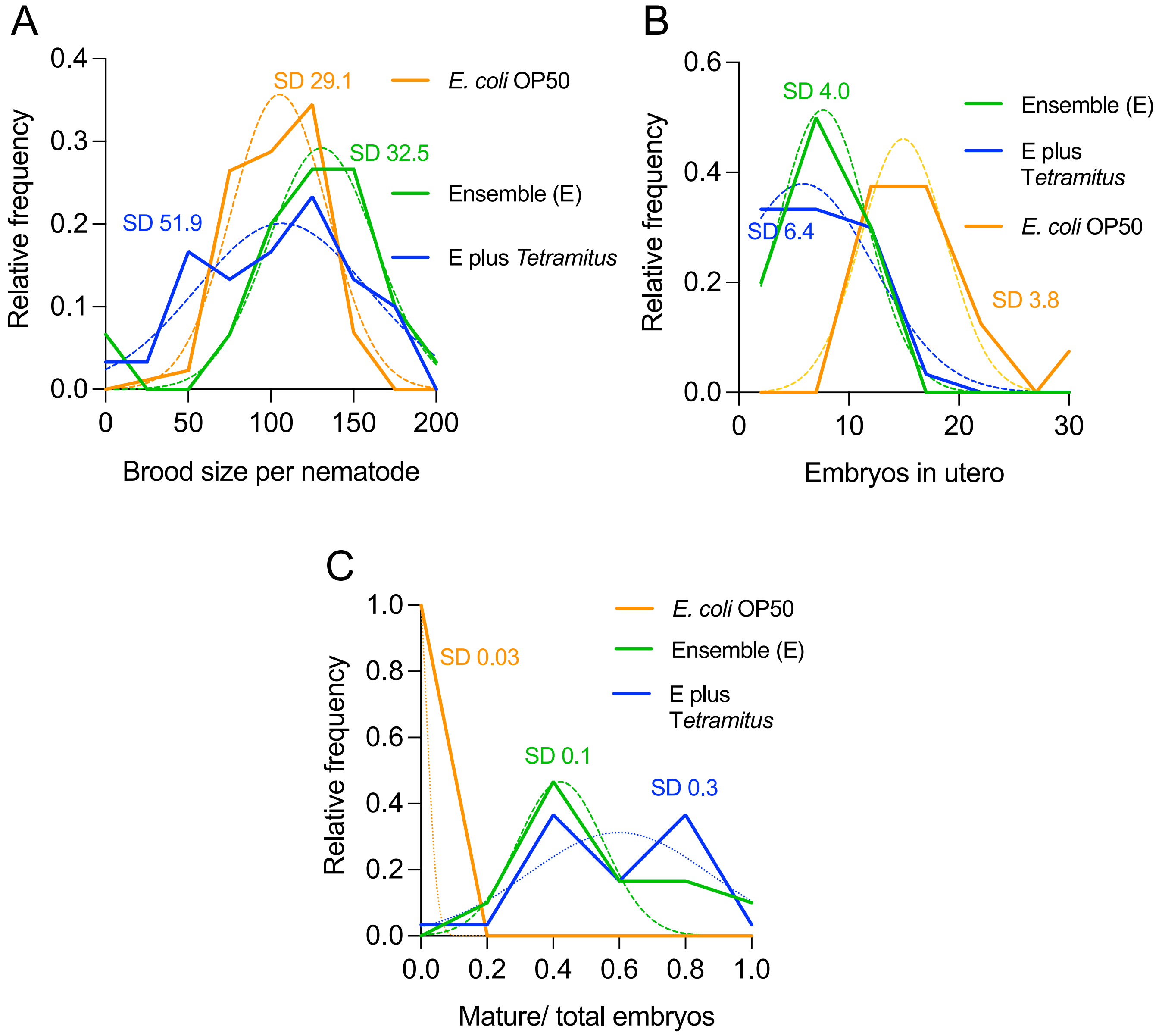

Figure S5. A. Relative frequency of every experimental value within the population of live progenies laid within a period of 72 hours by individual gravid adults. **B**. Relative frequency of data points in the population of ensembles with and without amoebae compared to *E. coli* OP50 of number of embryos in utero. **C**. Relative frequency of the ratio of embryos expressing *gfp* in the AWB and AWC neurons vs total embryos in utero. The standard deviation for the condition of ensembles and ensembles with amoeba and the comparison with *E. coli* OP50 is shown. Each experiment included three technical triplicates and at least three biological replicates. Complete statistical analyses are detailed in **Dataset 2**.

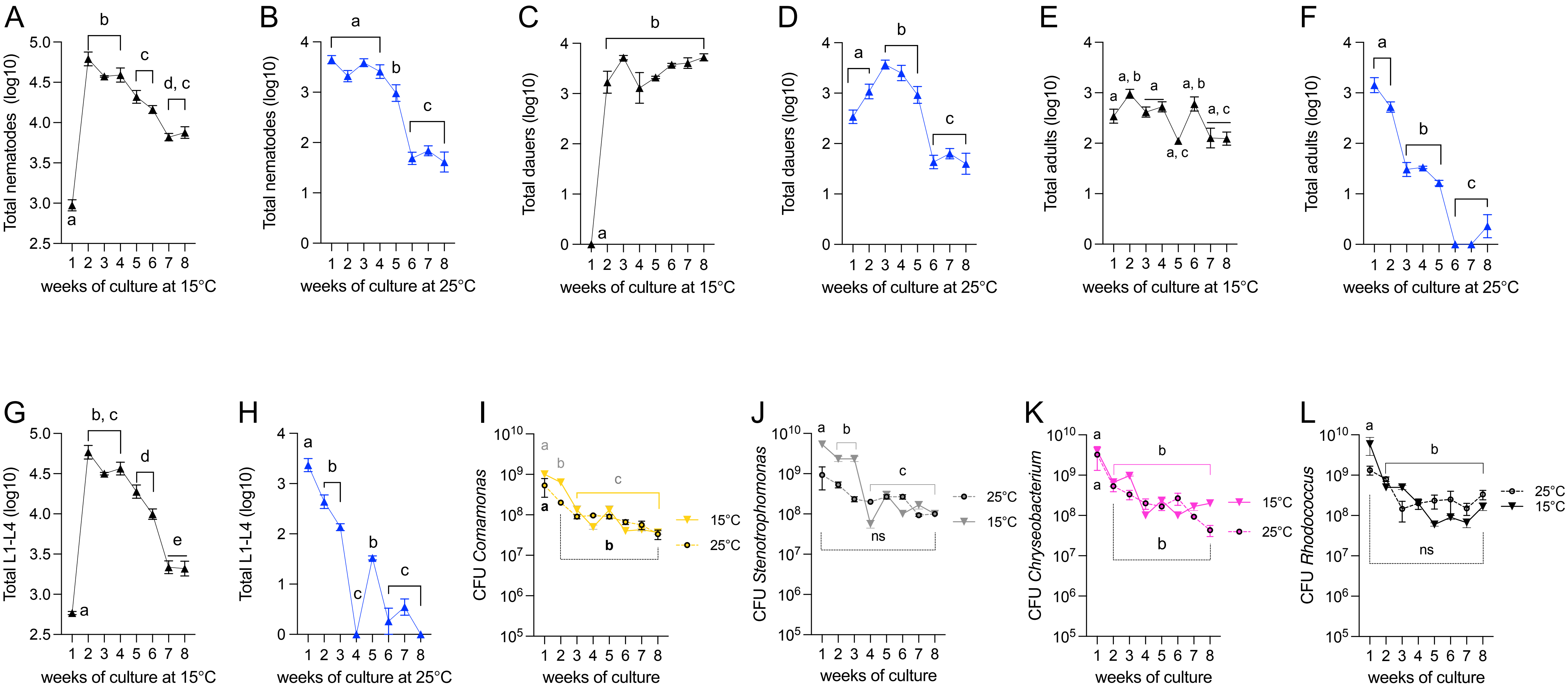

Figure S6. A-H. Total numbers of nematodes (**A, B**) and among those dauers (**C, D**), adults (**E, F**) and L1-L4 (**G, H**), in long term cultures at 15°C (**A, C, E, G**) and 25°C (**B, D, F, H**). **I-L**. CFU count of *Comamonas* (I), *Stenotrophomonas* (J), *Chryseobacterium* (K) and *Rhodococcus* (L), in the ensemble at 15°C and 25°C. Same letters denote no statistical differences, while different letters indicate statistically significant differences. Each experiment included three technical triplicates and at least three biological replicates. Complete statistical analyses are detailed in **Dataset 2**.
